# Present status and future directions – Managing discoloured teeth

**DOI:** 10.1111/iej.13711

**Published:** 2022-03-08

**Authors:** Bill Kahler

**Affiliations:** ^1^ School of Dentistry The University of Queensland Brisbane Queensland Australia; ^2^ The University of Queensland Oral Health Centre Herston Queensland Australia

**Keywords:** bleaching, carbamide peroxide, external cervical root resorption, hydrogen peroxide, sodium perborate, walking bleach technique

## Abstract

Managing tooth discolouration involves a range of different protocols for clinicians and patients in order to achieve an aesthetic result. There is an increasing public awareness in the appearance of their teeth and management of tooth discolouration may be inter‐disciplinary and involve both vital and nonvital teeth. Vital teeth can be easily treated with low concentration hydrogen peroxide products safely and effectively using an external approach and trays. For endodontically treated teeth, the walking bleach technique with hydrogen releasing peroxide products is popular. However, there is an association with external cervical root resorption with higher concentrations of hydrogen peroxide of 30%–35%. There are also regulatory considerations for the use of hydrogen peroxide in certain jurisdictions internationally. Prosthodontic treatments are more invasive and involve loss of tooth structure as well as a life cycle of further treatment in the future. This narrative review is based on searches on PubMed and the Cochrane library. Bleaching endodontically treated teeth can be considered a safe and effective protocol in the management of discoloured teeth. However, the association between bleaching and resorption remains unclear although there is likely to be a relation to prior trauma. It is prudent to avoid thermocatalytic approaches and to use a base/sealer to cover the root filling. An awareness expectations of patients and multidisciplinary treatment considerations is important in achieving the aesthetic result for the patient. It is likely that there will be an increasing demand for aesthetic whitening treatments. Bleaching of teeth has also become increasingly regulated although there are international differences in the use and concentration of bleaching agents.

## INTRODUCTION

Managing tooth discolouration involves a range of clinical protocols in order to achieve an aesthetic result. The aetiology of the discolouration needs to be considered as well as any underlying infection as there are different approaches for vital teeth when compared to endodontically treated teeth which involve either external or internal approaches to the tooth. Patient's expectations can also extend beyond just the colour of the tooth requiring prosthodontic rehabilitation. While this review will consider the topic broadly, the focus will primarily be concerned with endodontically treated teeth as trauma is a major aetiology of tooth discolouration (Hattab et al., 1999). Tooth discolouration can also be considered as intrinsic (staining) or extrinsic factors introduced by endodontic procedures and the materials used in root filling the tooth (Ahmed & Abbott, 2012; Hattab et al., 1999; Krastl et al., 2013; Plotino et al., 2008). Tooth bleaching involves hydrogen peroxide which is either applied directly or produced from a chemical reaction from sodium perborate or carbamide peroxide. Prior reviews on nonvital bleaching have been published (Attin et al., 2003; Plotino et al., 2008) but since they were published, the European Union published a Cosmetic Directive (2011/84/EU) which confirmed that the bleaching products with concentrations above 0.1% hydrogen peroxide are not permitted for use on people under the age of 18 years. This directive also limited dentists to using a maximum concentration of 6% hydrogen peroxide for bleaching protocols (Official Journal of the European Union Council Directive, 2011). While this application is limited to the European Union, similar regulations for bleaching are appearing internationally (Australian Dental Board, AHPRA). As this special edition of the International Endodontic Journal addresses the present status and future directions in management of discoloured teeth, the issue of bleaching must be considered not only for efficacy but in a potential new regulatory framework. The effectiveness of bleaching with lower concentrations of hydrogen peroxide and satisfying patient's expectations in a new regulatory framework are important considerations along with alternative treatments that involve prosthodontic alteration to the teeth with associated tooth loss. Discoloured teeth are known to impact on the quality‐of‐life issues for patients (Bonafé et al., 2021). There are also increasing aesthetic demands on clinicians to provide ‘the perfect smile’ (Pavicic et al., 2018).

## LITERATURE SEARCH

For the purpose of this review, a comprehensive literature search was undertaken using PubMed and the Cochrane Library. Different keyword combinations were used including ‘bleaching’, ‘outcomes’, ‘vital teeth’, ‘non‐vital teeth’, ‘walking bleach technique’, ‘hydrogen peroxide’, ‘sodium perborate’ and ‘carbamide peroxide’. The reference lists of the selected papers, previous review papers and the recent chapters in textbook were also assessed for relevant literature. Other search terms relevant for the review which may also involve periodontal and prosthodontic rehabilitation, included ‘crown outcomes’, ‘crown survival’, ‘crown complications’ and ‘crown lengthening’.

## AETIOLOGY OF TOOTH DISCOLOURATION

Tooth discolouration can be considered intrinsic, extrinsic or a combination of both (Hattab et al., 1999). The colour of the tooth is primarily determined by the colour of the dentine (Ten Bosch & Coops, 1995) and influenced by intrinsic and extrinsic impacts (Watts & Addy, 2001). The main aetiological factors are presented in Table 1 (Adapted from Plotino et al. 2008 and Setzer, 2020).

**TABLE 1:** Intrinsic and extrinsic causes for discolouration of teeth

Extrinsic causes	Intrinsic causes
Dietary: Wine, coffee, tea, carrots, oranges, liquorice, chocolate, betel nut	Systemic causes a) Drug related, e.g. tetracycline, minocycline used in RET b) Metabolic, e.g. congenital erythropoietic porphyria, cystic fibrosis of the pancreas, hyperbilirubinemia, thalassemia, amelogenesis imperfecta, dentinogenesis imperfecta
Tobacco	Local causes a) Pulp necrosis, e.g. traumaIntrapulpal haemorrhage b) Calcification of the canal following trauma c) Remaining pulp tissue remnants following endodontic treatment d) Root canal irrigants e) Root canal sealers f) Repair materials and secondary effects involving interactions with NaOCl and/or blood g) Endodontic materials such as amalgam, coronal leakage or MTA and triple or double antibiotic pastes used in REPs h) Coronal filling materials including temporary restorations, e.g. IRM i) Root resorption j) Aging k) Fluorosis l) Caries
Mouth rinses, e.g. Chlorhexidine	Resorptions. Invasive cervical – pinkish colour
Plaque, e.g. Chromogenic bacteria	

Abbreviations: MTA, mineral trioxide aggregate; REPs, regenerative endodontic protocols.

There are known causes for discolouration of endodontically treated teeth which include root canal irrigants especially the combination of chlorhexidine when used in combination with sodium hypochlorite that produces an insoluble dark brown precipitate, intracanal medicaments, antibiotic pastes, mineral trioxide aggregate (MTA) used in perforation repairs and regenerative endodontic treatments, endodontic sealers and provisional restorations. Root canal materials that incorporate trioxide as a filler and radiopoacifier are particularly implicated (Walsh & Athanassiadis, 2007). AH 26 (Dentsply De Trey) contains silver which can corrode and produce a grey/black discolouration (Allan et al., 2001). The newer material, AH Plus (Dentsply De Trey) contains zirconium oxide as the radiopacifier and has better colour stability over time (Walsh & Athanassiadis, 2007). Therefore. Ahmed and Abbott (2012) advocated for preventive measures to be taken to reduce the risk of discolouration. These measures include an appropriate access cavity, flushing medicaments with saline after their use, placing endodontic materials below the gingival margin and avoiding metallic restorations in teeth as well as avoiding the potential for coronal leakage if resin composites are placed too soon after the bleaching process. Thomson et al. (2012) in a study that analysed coronal colour change over a 12‐month time frame suggest alternative medicaments and sealers to Ledermix (Lederle Laboratories) and AH Plus when there were aesthetic considerations. Krastl et al. (2013) also advised careful application of endodontic materials carefully where there are aesthetic considerations. Furthermore, aesthetic considerations should be considered in conjunction with biological and functional requirements of treatment.

Teeth that have sustained a traumatic incident with associated intrapulpal haemorrhage often discolour as blood components diffuse into dentinal tubules. Otherwise the haemolysis of erythrocytes releases iron (Marin et al., 1997) which often results in brown/reddish and black discolourations especially following a traumatic event that results in pulp necrosis (Setzer, 2020). An important consideration is that teeth that maintain vitality may regain normal colour (Andreasen & Kahler, 2015). In root filled teeth, inadequate cleaning of the access cavity and pulp chamber by leaving necrotic pulp remnants in pulp horns or failure to adequately remove root filling pastes can also cause discolouration after root canal treatment.

Tooth discolouration has also been associated with regenerative endodontic protocols (REPs) that utilize triple antibiotic pastes (TAPs) that contain minocycline and MTA as an intracanal barrier over an induced blood clot to the level of the cemento‐enamel junction (CEJ) (Kahler et al., 2014; Kahler & Rossi‐Fedele, 2016).

## HISTORY OF BLEACHING TEETH

Bleaching of nonvital discoloured teeth was first described by Truman, 1864. Harlan (1884) described the use of hydrogen peroxide for bleaching pulpless teeth whilst Abbott (1918) used Superoxol 30% hydrogen peroxide in combination with electric light rays. Prinz (1924) described heated solutions of sodium perborate and Superoxol in the pulp space. The ‘walking bleach technique’ was developed using sodium perborate and water placed into the pulp chamber which was sealed into the root canal space (Salvas, 1938; Spasser, 1961). Nutting and Poe (1963) improved the whitening efficacy by replacing the water with 30%–35% hydrogen peroxide. Many different bleaching agents have been used and their type, concentration, whether heat is also applied, and efficacy are listed in Table 2. Also, in the 1960s an orthodontist observed whiter teeth when 10% carbamide peroxide was used in a tray for the treatment of gingivitis (Haywood, 1991). Haywood and Heymann (1989) described the use of bleaching trays and 10% carbamide peroxide for lightening tooth colour in vital teeth.

**TABLE 2:** *In vivo* studies reporting the success rate of internal bleaching of endodontically treated teeth

Author(s)	Number	Bleaching agent	Review (years)	Success	Complications/comments
Brown (1965)	80	Thermocatalytic: 30% H_2_O_2_, followed by WBT: 30% H_2_O_2_	1–5	75% success (39% complete, 46% partial); 25% failure (17.5% no improvement, 7.5% refractory discolouration	Severely discoloured teeth have less successful outcomes vs. moderate and slight discolouration (75%–90%−100%)
Tewari and Chawler (1972)	19	Thermocatalytic: 30% H_2_O_2_	5	95% success 5% failure	The only failure was successfully bleached again
Chandra and Chawla (1972)	230	15 different techniques	1	95% success 5% failure	Failures associated with insufficient fillings
Howell (1980)	41	Thermocatalytic: 30% H_2_O_2_, followed by WBT: 30% H_2_O_2_	Immediate assessment post‐ bleaching	97% success (90% complete, 7% partial) 3% failure	Tooth that failed was discoloured for 40 years
Howell (1981)	339	Thermocatalytic: 30% H_2_O_2_, followed by WBT: 30% H_2_O_2_	1	97% success (53% complete, 44% partial, 3% failure)	Discoloured tooth had a leaking filling and bleached again successfully
	19		2	100% success (42% complete, 58% partial)	Colour regression in 50% after I year.
Feiglin (1987)	20	Thermocatalytic: 130 vol H_2_O_2_ followed by WBT ¾ H_2_O_2_ and 130 vol H_2_O_2_	6	45% success 55% failure	Better outcomes for younger patients. Aesthetic regression with time
Friedman et al., 1988	58	Thermocatalytic 30% H_2_O_2_, WBT 30% H_2_O_2_, Thermocatalytic: 30% H_2_O_2_, followed by WBT: 30% H_2_O_2_	8	50% success 29% acceptable 21% failure	Most failures occurred between 2 and 8 years post‐bleaching
Holmstrup et al. (1988)	95 69	Thermocatalytic: 30% H_2_O_2_, followed by WBT: 30% H_2_O_2_	Immediate assessment post‐ bleaching 3	63% success (63% good, 26% acceptable) 10% failure 79% success (49% good, 30% acceptable) 20% failure	Three teeth with transient pain
Anitua et al. (1990)	258	WBT: SP + 110 vol H_2_O_2_	4	100% (90% complete, 10% partial) success	All teeth were tetracycline stained and elective RCT
Aldecoa and Mayordomo (1992)	534	WBT	6	Stable results	All teeth were tetracycline stained and elective RCT
Waterhouse and Nunn (1996)	21	WBT 30% H_2_O_2_ + SP granules	1.5	83% success	Teeth discoloured with time
Abou‐Rass (1998)	112	WBT: SP + 30% H_2_O_2_	3–15	93% success 7% failure	All teeth were tetracycline stained and elective RCT
Glockner et al. (1999)	86	WBT: SP + 30% H_2_O_2_	5	84% success in ideal cases	Ideal cases were intact crowns
Bizhang et al. (2003)	61	EC 10% CP; WBT SP + 3% H_2_O_2_, EC + WBT 10% CP	0.5	EC + 10% CP was as effective as WBT (SP + 3% H_2_O_2_)	The EC approach reduced bleaching time by 50%
Amato et al., 2006	35	Mixture of SP + 120 vol H_2_O_2_	16	69.9% success 37.1% failure	No incidence of resorption in this long‐term study
Abbott and Heah (2009)	255	WBT 35% H_2_O_2_ + SP powder	0.5–5	Success 100% (Good 87.1% Acceptable 12.9%)	Rebleaching was associated with restoration failure
Badole et al. (2013)	3	35% CP	0.4–1	100% success	
Bersezio et al. (2018a)	38	35% H_2_O 37% CP	0.5	Colour improved in both groups	
Amato et al. (2018)	60	EC + WBT 10% CP	25	85% success	No incidence of resorption in this long‐term study
Lise et al. (2018)	17	9: SP + 20% H_2_O_2_ 8: I‐O 10% CP	1	Significant improvement	Stable at 1 year review

Note

Adapted from Attin et al. (2003) and Plotino et al. (2008) and extended to 2021. Copyright clearance was obtained.

Abbreviations: CP, Carbamide peroxide; EC, Extra‐coronal; H_2_O_2_, Hydrogen peroxide; I‐O. Inside‐Outside technique; SP, Sodium perborate; WBT, Walking bleach technique.

## TOXICITY OF BLEACHING AGENTS

Hydrogen peroxide exposure to the gingiva can induce epithelial damage (Martin et al., 1968) with 25%–50% of patients in clinical trials experiencing gingival irritation with the use of custom‐made trays (Bruzell et al., 2013; Leonard et al., 1997). There may also be an increase in tooth sensitivity with vital bleaching (Leonard, 1998). Tooth sensitivity is usually mild to moderate and transient lasting only a few days (Jorgensen & Carroll, 2002; Leonard et al., 1997; Pohjola et al., 2002) but longer‐term discomfort has been reported (Leonard et al., 1997; Tam, 1999). Laboratory studies have reported that hydrogen peroxide penetrates enamel and dentine to enter the pulp space (Thitinanthapan et al., 1999). The degree of penetration depended on the concentration of the hydrogen peroxide (Gökay et al., 2000). However, structural pulp damage was not shown in human teeth exposed to 35% hydrogen peroxide *in vivo* that were extracted and examined histologically (Baumgartner et al., 1983; Cohen & Chase, 1979; Robertson & Melfi, 1980). Hydrogen peroxide is only considered a low risk to produce local carcinogenic outcomes with the IARC (1999) concluding there was inadequate evidence in humans for the carcinogenicity of hydrogen peroxide (Dahl et al., 2019).

## TREATMENT PLANNING OPTIONS

The discoloured tooth/teeth can be a great challenge to obtain pleasing aesthetic outcomes affecting the ‘smile zone’. The causes and extent of the discolouration must be assessed. The vitality of the pulp, prior endodontic treatment, signs and symptoms of infection will determine whether external or internal bleaching is advised. The colour of the root, the thickness of the gingiva and the tooth form are also important determinants on treatment choice.

Treatment options for improving the colour of teeth can include:

1. External bleaching – At‐home external bleaching, power bleaching, abrasion techniques, lasers.
2. Internal bleaching – Walking bleach, internal/external bleaching, thermocatalytic bleaching.
3. Prosthodontic options – resin composite and ceramic veneers, crowns.

Periodontal considerations such as root colour, tooth shape and form should be considered in the treatment planning.

## EXTERNAL BLEACHING – AT‐HOME EXTERNAL BLEACHING, POWER BLEACHING, ABRASION TECHNIQUES, LASERS

Vital tooth bleaching is a technically easy and low‐cost method to improve tooth colour. Nightguard vital bleaching is a safe procedure with satisfactory retention of shade with only mild and transient side effects disappearing within days of treatment completion (Leonard, 2000). There are increased demands from patients to improve the colour of vital, natural teeth (Maran et al., 2018; Wilson et al., 2004). Whitening toothpastes are popular with high public demand (Alkahtani et al., 2020). Studies also reveal that many adults are dissatisfied with the appearance of their teeth (Alkhatib et al., 2004; Odioso et al., 2000; Qualtrough & Burke, 1994). There are three approaches generally used that include (1) in‐office and professionally administered, (2) at home and professionally administered or (3) commercially available and self‐administered procedures that use products based on hydrogen peroxide or carbamide peroxide (Dahl & Pallesen, 2003; de Geus et al., 2016). The in‐office approach allows for professional application to avoid soft‐tissue damage and potentially provide more rapid aesthetic outcomes (Alomari & Daraa, 2010; Gurgan et al., 2010; Henry et al., 2013). Power bleaching approaches activate catalytic decomposition of the peroxide products by heat or light to enhance the release of oxygen‐based free radicals (Kashima‐Tanaka et al., 2003; Maran et al., 2018). Light activation sources have included light‐emitting diodes, plasma arc lamps, halogen lamps and lasers (Ontiveros, 2011). However, commercially claimed benefits of light in accelerated colour improvements was not confirmed in a systematic review and meta‐analysis (Maran et al., 2018). A further systematic review and meta‐analysis reported that the use of lower concentration hydrogen peroxide products produced similar colour changes to that of higher concentration products with less risk and intensity of bleaching sensitivity (Maran et al., 2020). The use of trays and peroxide bleaching is safe to enamel surfaces (Mielczarek et al., 2008) although other studies have shown damage with a significant decrease in enamel hardness (Akal et al., 2001; Araujo et al., 2013). In a recent review, new whitening products and technologies including nano‐additives and different carrier systems may maximize the bleaching process and minimize structural enamel damage (Alkahtani et al., 2020). However, dental bleaching can also be associated with demineralization of remineralized carious lesions as, while the colour of the arrested lesion was improved, the risk of demineralization was increased, especially when associated with adhesive restorations (Al‐Angari et al., 2019).

Micro‐abrasion uses peroxide products for shallow intrinsic stains and superficial irregularities in the enamel (Gupta et al., 2017). Successful long‐term follow‐up has been reported (Sundfeld et al., 2014).

**FIGURE 1:**
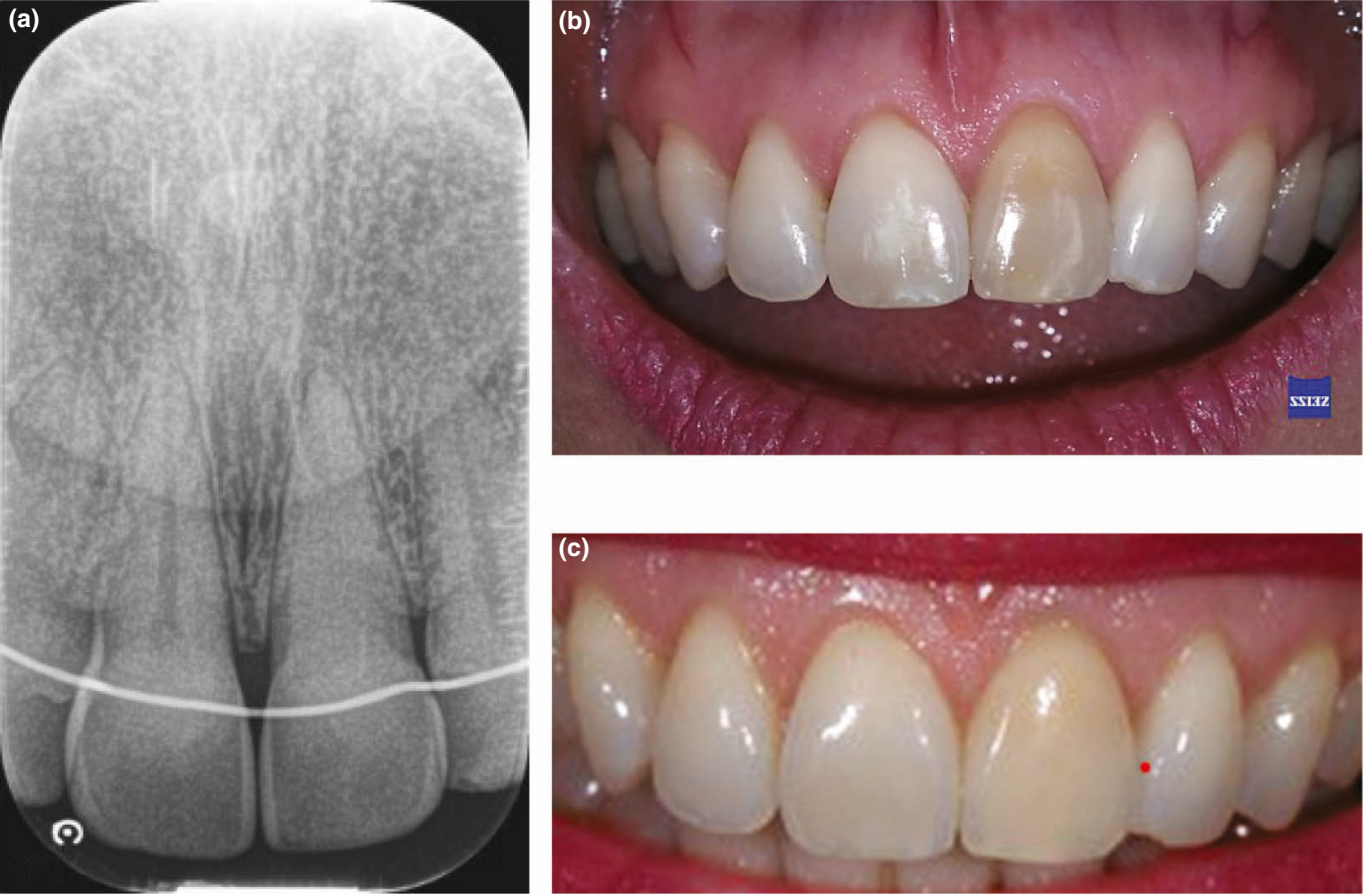
(a) A periapical radiograph of a left maxillary central incisor with extensive obliteration of the canal. The tooth was asymptomatic and nonresponsive to cold and electric pulp sensibility testing. Radiographically, there was no evidence of pathosis. (b) A preoperative photo showing discolouration. (c) The tooth was managed with external bleaching protocols using 6% hydrogen peroxide and customized trays (pola day/pola night; SDI Ltd.). The aesthetic outcome met the patient's expectation and improved the colour of all teeth in the smile zone. Courtesy of Dr Michael Lewis

**FIGURE 2:**
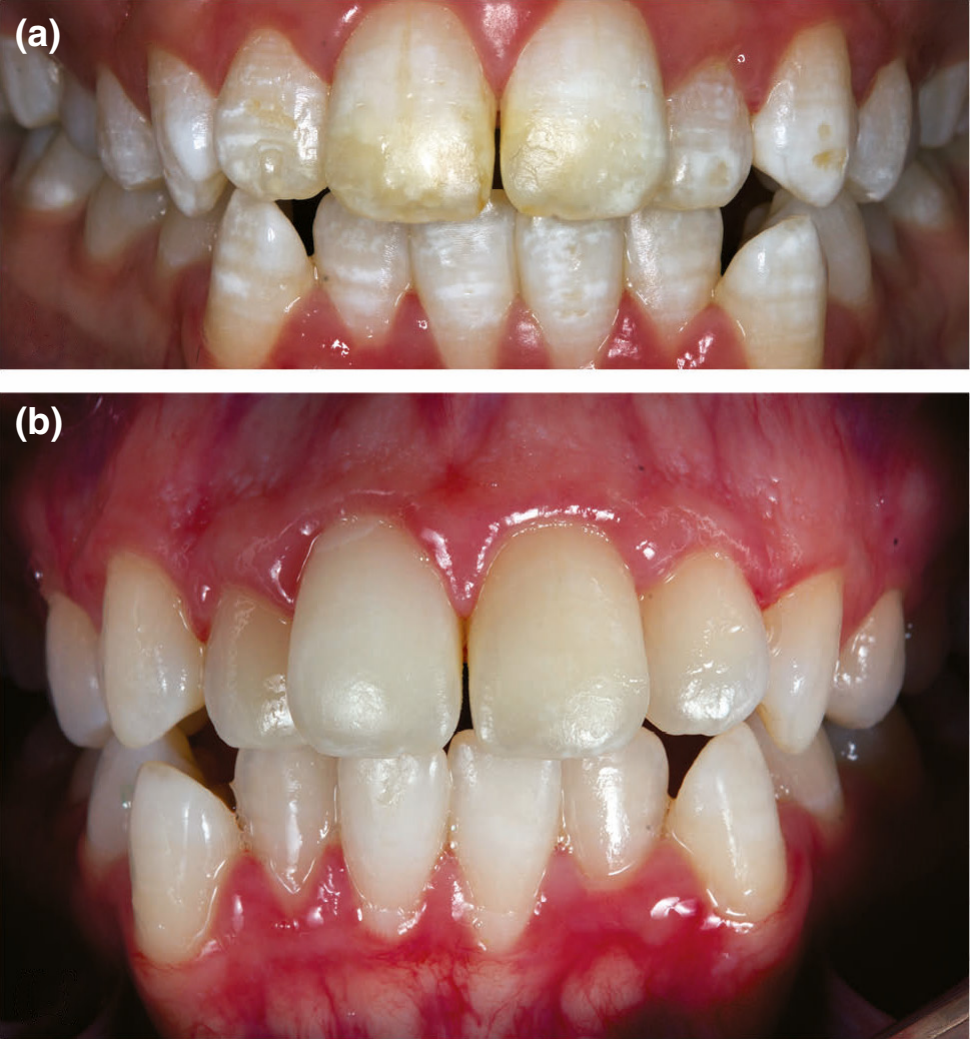
(a) A photograph of an 18‐year‐old girl with moderate fluorosis. (b) A post‐treatment photograph. The teeth received a single visit in‐office bleaching with Philips ZOOM (Philips Oral Healthcare) Light Activator using Zoom White Speed gel (25% Hydrogen peroxide). Superficial stains and plaque were first removed using a prophy paste in a rubber cup. The lips were protected with sunblock cream, prior to placement of lip retractors. Isolation of the gingiva was achieved by cotton rolls placed in the vestibule and light‐cured resin barrier. The teeth received a total of 30 min application of bleach illuminated with ZOOM lamp on High intensity (190 mW/cm^2^) for 15 min, followed by medium intensity (120 mW/cm^2^) for 15 min. One week later, resin infiltration treatment was accomplished using ICON system for three etching cycles, followed by two resin infiltration cycles. Courtesy of Professor Zafer Cehreli

**FIGURE 3:**
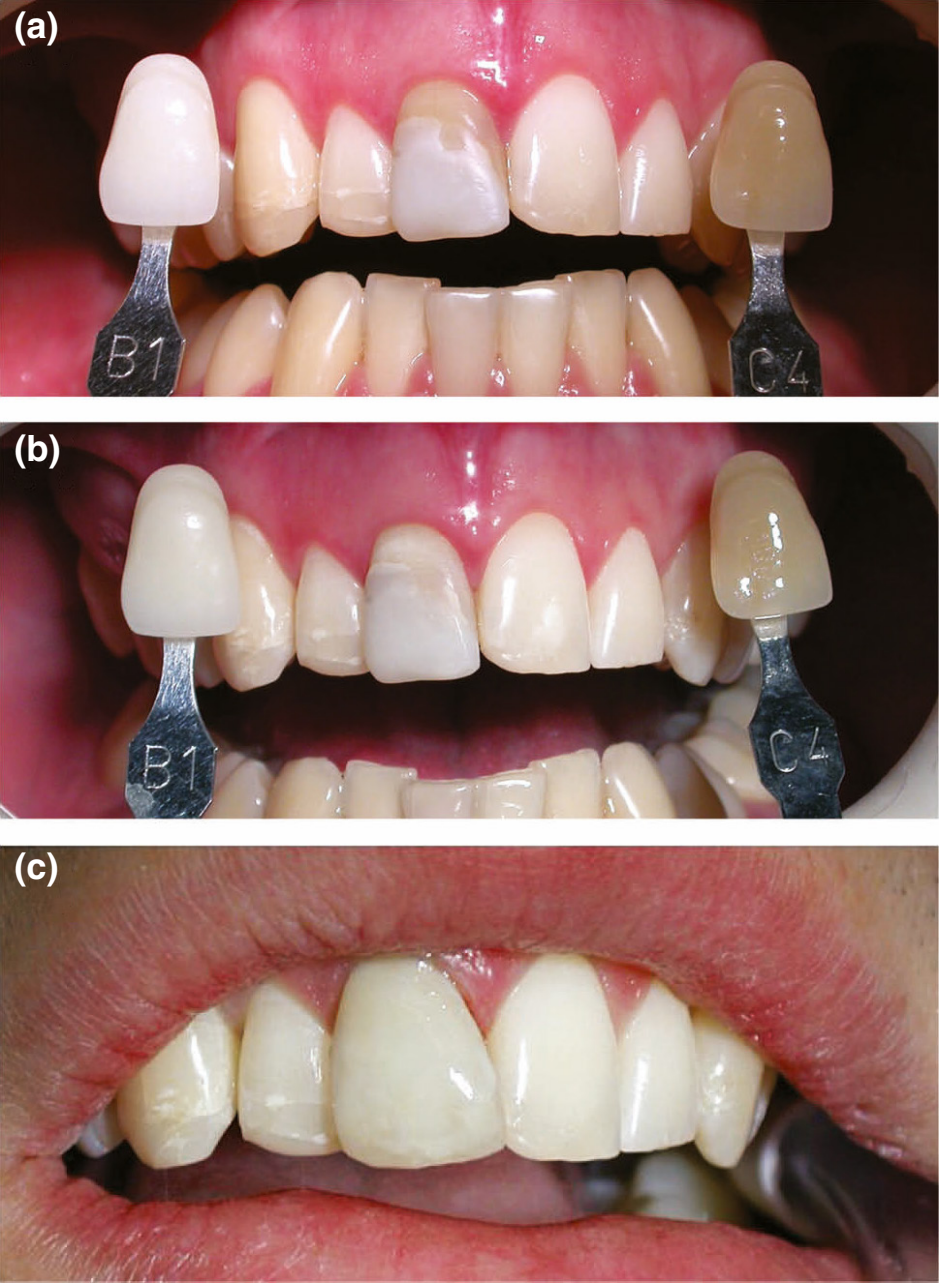
(a) A photograph revealing a discoloured right maxillary central incisor and mismatch rein composite restoration (b) A photograph after treatment of 30 min with a KTP laser (Smartlite; Deka) The laser is used to lighten the crown colour of the enamel so translucent resin composite materials can be used with less or no need for opaquing agents which block light transmission through teeth. (c) A photograph after replacement of the resin composite. Courtesy of Professor Laurence Walsh

It is not the purpose of the current review to evaluate vital bleaching techniques comprehensively. Further information is available from existing papers (Alkahtani et al., 2020; Joiner, 2006). Studies indicate that 1%–16% of teeth undergoing calcific metamorphosis will require endodontic treatment so nonendodontic whitening of these teeth needs to be considered (Amir et al., 2001). Therefore, external bleaching is indicated in the management of discoloured teeth (Figures 1, 2, 3).

## INTERNAL BLEACHING – WALKING BLEACH, INTERNAL/EXTERNAL BLEACHING, THERMOCATALYTIC BLEACHING

### Bleaching agents for whitening of root filled teeth

Tooth bleaching utilizes hydrogen peroxide as the active agent in an oxidative process that degrades larger discoloured compounds and stains. Hydrogen peroxide can be applied directly or produced in a chemical reaction from sodium perborate or carbamide peroxide (Budavari et al., 1989). Hydrogen peroxide releases free radicals (Gregus & Klaassen, 1995), reactive oxygen molecules and hydrogen peroxide anions (Cotton & Wilkinson, 1972). Tooth bleaching involves the chemical alteration of light‐absorbing or light‐reflecting properties and structure of enamel and dentine stains. The reactive molecules break down the long‐chained, dark coloured chromophore molecules and split the double bonds into smaller, less coloured and more diffusible molecules (Dahl & Palleson, 2003). The change in pigment configuration and size alters the wavelength of the reflected light with the result is the stain appearing lighter in colour that is seen as whitening (Frysh et al., 1995; Nathoo, 1997). However, after the bleaching procedure, the double bonds can reform the chromogen molecules leading to a shade rebound effect (Setzer, 2020).

The decomposition of hydrogen peroxide into active oxygen products is accelerated by heat, addition of sodium hydroxide and light (Chen et al. 1993, Hardman et al. 1985). Hydrogen peroxide‐releasing bleaching agents are chemically unstable and should be stored in a dark, cool place or refrigerator (Plotino et al., 2008). Hydrogen peroxide has a low molecular weight which allows it to penetrate dentine, release oxygen to break down the double bonds of organic and inorganic substances within dentinal tubules and stained dentine (Seghi & Denry, 1992). The thermocatalytic technique used to be considered the best approach because of the increased reactivity of the technique with application of heat by either special lamps or hot instruments (Abramson et al. 1966; Boksman et al. 1984; Brown, 1965; Grossman et al., 1988; Grossman, 1940; Howell, 1980; Hulsmann, 1993; Ingle, 1965; Kopp, 1973; Tewari & Chawla, 1972; Weine, 1982). Studies relating to the efficacy of bleaching *in vivo* and *in vitro* are set out in Tables 2 and 3.

**TABLE 3:** *In vitro* studies on the efficacy of nonvital bleaching

Authors	Bleaching agent	Success	Complications/comment
Ho and Goerig (1989)	Group 1: New SP + 35% H_2_O_2_	93% success	Colour regression after 6 months was found in 4% of cases
	Group 2: New SP + 1‐year old 35% H_2_O_2_	73% success	
	Group 3: New SP + distilled H_2_O	53% success	
	Group 4: Old SP + distilled H_2_O	53% success	
Casey et al. (1989)	Group 1: dentinal etching of the pulp chamber + WBT 30% H_2_O_2_ + SP Group 2: No etching WBT: 30% H_2_O_2_+SP	No statistical differences between the 2 groups	None
Warren et al. (1990)	35% H_2_O_2_, SP, 35% H_2_O_2_+ SP	35% H_2_O_2_ + SP was more effecting in improving crown and root colour	Effectiveness of the IRM cervical seal was questioned as root colour improved whether IRM placed at CEJ and 2 mm below CEJ
Rotstein et al. (1991)	SP + 30% H_2_O_2,_ SP + 3% H_2_O_2_, SP + H_2_O	No significant differences between the groups after 14 days	SP + H_2_O was recommended to reduce the risk of cervical resorption
Weiger et al. (1994a)	30% H_2_O_2_ + varying types of SP mixed with H_2_O or as a gel	Success rates ranged between 46% and 77%. Shorter bleaching times than 3–7 days were effective	
Weiger et al. (1994b)	SP + monohydrate (MH), trihydrate (TRH) or tetrahydrate (TH) was mixed with 35% H_2_O_2_ or H_2_O	Almost all teeth of the experimental groups showed leakage of H_2_O_2_ and on the form of sodium perborate used	Risk of cervical resorption by using SP tetrahydrate + H_2_O
Lenhard (1996)	10% CP	Most significant colour change occurred in the incisal section of the crown, followed by the middle and then cervical sections	The observed tooth colour change was dependant on the bleaching time, the specific bleached section, and the initial colour
Leonard et al. (1998)	5%–10%–16% CP	Higher concentrations of CP resulted in quicker colour change	Lower concentrations of CP took longer to bleach teeth and achieved the same result with extra time
Horn et al. (1998)	35% H_2_O_2_+SP Sterile H_2_O + SP	Teeth bleached with35% H_2_O_2_+SP were significantly lighter	Presence or absence of the smear layer did not influence the outcome of bleaching
Marin et al. (1998)	30% SP	Most efficient removal of staining occurred with 30% H_2_O_2_ with 75% CP as effective.	All bleaching agents were effective in 3 days
Jones et al. (1999)	35% H_2_O_2_, 10% and 20% CP	20% CP resulted in the greatest colour change	None
Ari and Üngör (2002)	Group 1: SP trihydrate + H_2_O Group 2: SP trihydrate + H_2_O Group 3: SP + tetrahydrate + H_2_O Group 4: SP trihydrate + H_2_O_2_ Group 5: SP trihydrate + H_2_O_2_ Group 6: SP tetrahydrate + H_2_O_2_	No statistically significant differences between the groups	None SP can be successfully mixed with H_2_O rather than H_2_O_2_ for effective bleaching
Joiner et al. (2004)	6% H_2_O_2_	H_2_O_2_ has no significant effect on microhardness of enamel and dentine	None
Camps et al. (2007)	20% H_2_O_2_ gel	Diffusion of H_2_O_2_ was higher for young teeth compared to older teeth.	Optimal renewal time for young teeth was 33 h
Yui et al. (2008)	WBT: Group 1. SP + distilled H_2_O Group 2. SP + 10%CP Group 3. SP + 35%CP	SP associated with CP was more effective than H_2_O	
Gökay et al. (2008)	10–17–37% CP or a mixture of SP + 30% H_2_O_2_	Peroxide penetration of gels was significantly lower than SP = H_2_O_2_	CP gels may carry less risk for resorption
Cardoso et al. (2013)	37% CP gel with and without activation, 35% H_2_O_2_ with and without activation	Ultrasonic activation of bleaching agents during internal bleaching was no more effective than without activation	
Hansen et al. (2014)	35% H_2_O_2_ and delayed restoration	Bleaching had a detrimental effect on bond strengths	Restoration of bleached teeth should be delayed for 1–2 weeks
Feiz et al. (2014)	45% CP gel, 45%SP + CP, SP + distilled H_2_O	CP gel and CP + SP gel significantly better than SP + H_2_O	Teeth tested were stained with resin‐based sealer
Sağlam et al. (2015)	WBT: SP + Nd:YAG laser, SP + diode laser, SP alone	Laser application especially Nd:YAG laser significantly increased bleaching efficacy	Enamel surface structure was not affected
Zoya et al. (2019)	SP + distilled H_2_O; SSPC + distilled H_2_O, SP + 30% H_2_O_2_, SSPC + 30% H_2_O_2_, 30% H_2_O_2_, distilled H_2_O	Extraradicular peroxide release from SSPC was not significantly different from SP	Lower concentrations of H_2_O_2_ should be used if used in conjunction with SP or SSPC
Papadopoulos et al., 2021	35% H_2_O_2_, 35% H_2_O_2_ with and without Er, Cr:YSGG laser on different power settings	Er, Cr:YSGG laser irradiation increased lightness only after the first bleaching session and after the second session was not different to the control group irrespective of the laser power settings	

Note

Adapted from Plotino et al. (2008) and extended to 2021.

Abbreviations: CP, carbamide peroxide; EC, extra‐coronal; H_2_O_2_, hydrogen peroxide; SP, sodium perborate; SSPC, sodium percarbonate; WBT, walking bleach technique.

Carbamide peroxide (CH_6_N_2_O_3_) breaks down into carbamide and hydrogen peroxide in an aqueous solution. It also produces urea (Budavari et al., 1989) which has a high pH that enhances the bleaching effect (Sun, 2000). Carbamide peroxide crystals and powder contain H_2_O_2_ in an approximate concentration of 35%. At 35%, there is slow extraradicular diffusion rates when compared to hydrogen peroxide and sodium perborate (Lee et al., 2004; Lim et al., 2004). Carbamide peroxide in contact with dentine releases oxygen products for 40–90 min in comparison with hydrogen peroxide where the release is more instantaneous (Nathoo, 1997).

Sodium perborate (NaBO_3_) is commercially available as a stable dry powder or gel in monohydrate, trihydrate and tetrahydrate forms that have varying oxygen contents. The bleaching efficacy is dependent on the oxygen content (Weiger et al., 1994a). The perborate ion comprises 95% of the molecule and provides approximately 10% of the available oxygen. The H_2_O_2_ released generates different radicals or ions depending on the pH value, light, temperature, co‐catalysts and addition of metallic reaction products (Feinman et al., 1991; Goldstein & Garber, 1995). Bleaching is effective in an alkaline environment with the release of perhydroxyl radicals (Goldstein & Garber, 1995). Hydrogen peroxide release from sodium perborate reaches peak concentration within 72 h and plateaus at 3 days (Tran et al., 2017). It is considered safer than hydrogen peroxide for intracoronal bleaching (Setzer, 2020).

A recent systematic review and meta‐analysis concluded that carbamide peroxide, hydrogen peroxide and sodium perborate all have a significant bleaching effect on discoloured root filled teeth. However, the efficacy of carbamide peroxide, hydrogen peroxide and hydrogen peroxide combined with sodium perborate was better than sodium perborate used as the sole bleaching agent (Frank et al., 2022).

In Europe there are regulatory issues with the European Union producing a Cosmetic Directive (2011/84/EU) advising bleaching products should not release more than 6% of hydrogen peroxide and were not to be used on people under the age of 18 years. However, there does appear to be a degree of latitude where the treatment is considered safe and necessary by the dentist (Dahl et al., 2019).

### Walking bleach technique

**FIGURE 4:**
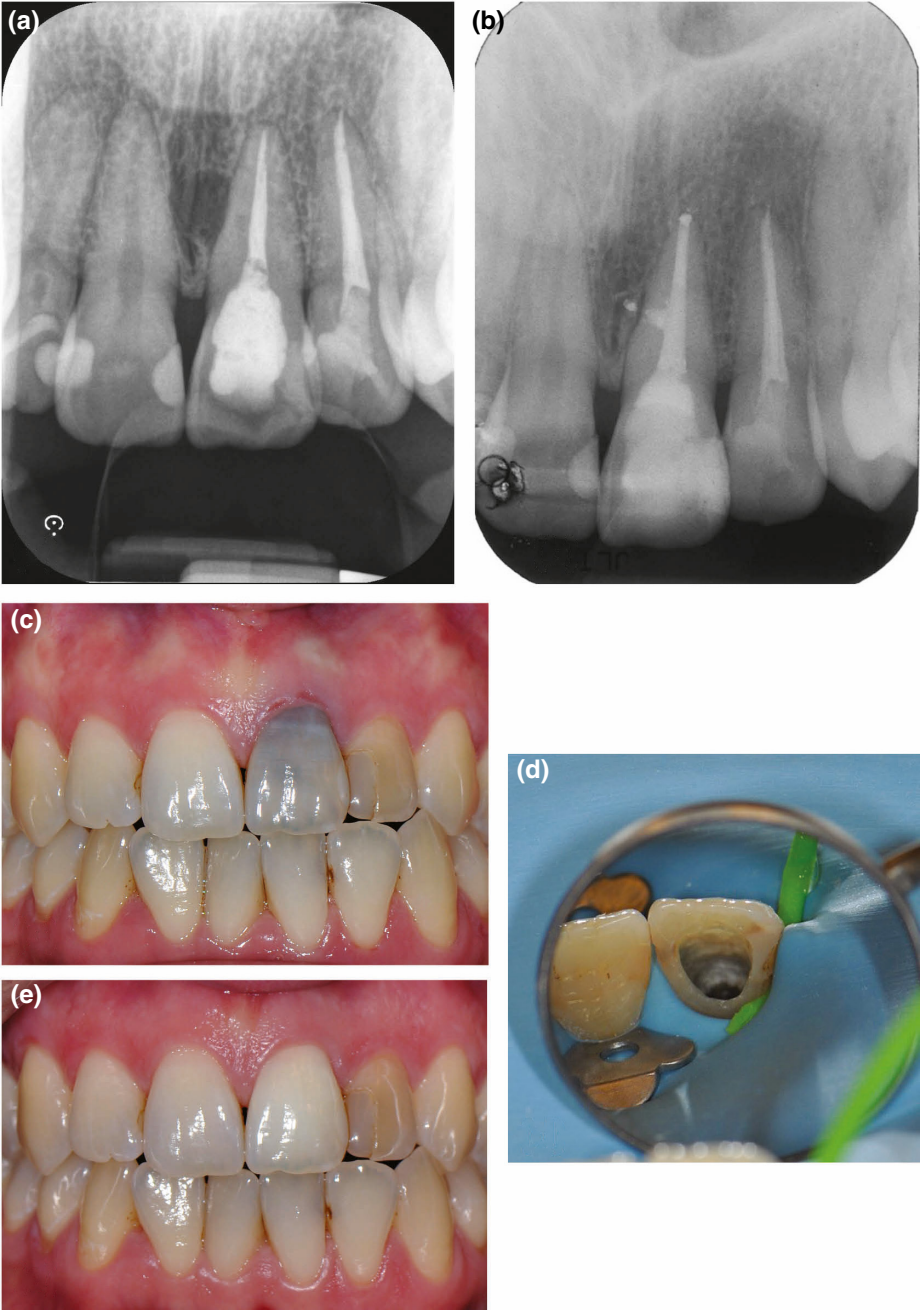
(a) A preoperative radiograph of a root filled left maxillary central incisor with pathos. (b) The tooth was successfully retreated. (c) A photograph showing the extent of discolouration. (d) A photograph of the open assess cavity. Bleaching utilized the walking bleach technique with sodium perborate (Endoprep bleach; PDS) mixed with water. (e) A post‐treatment photograph immediately post‐bleaching showing the bleached tooth having a lighter colour than the adjacent teeth. Courtesy of Dr Tom Fenelon

**FIGURE 5:**
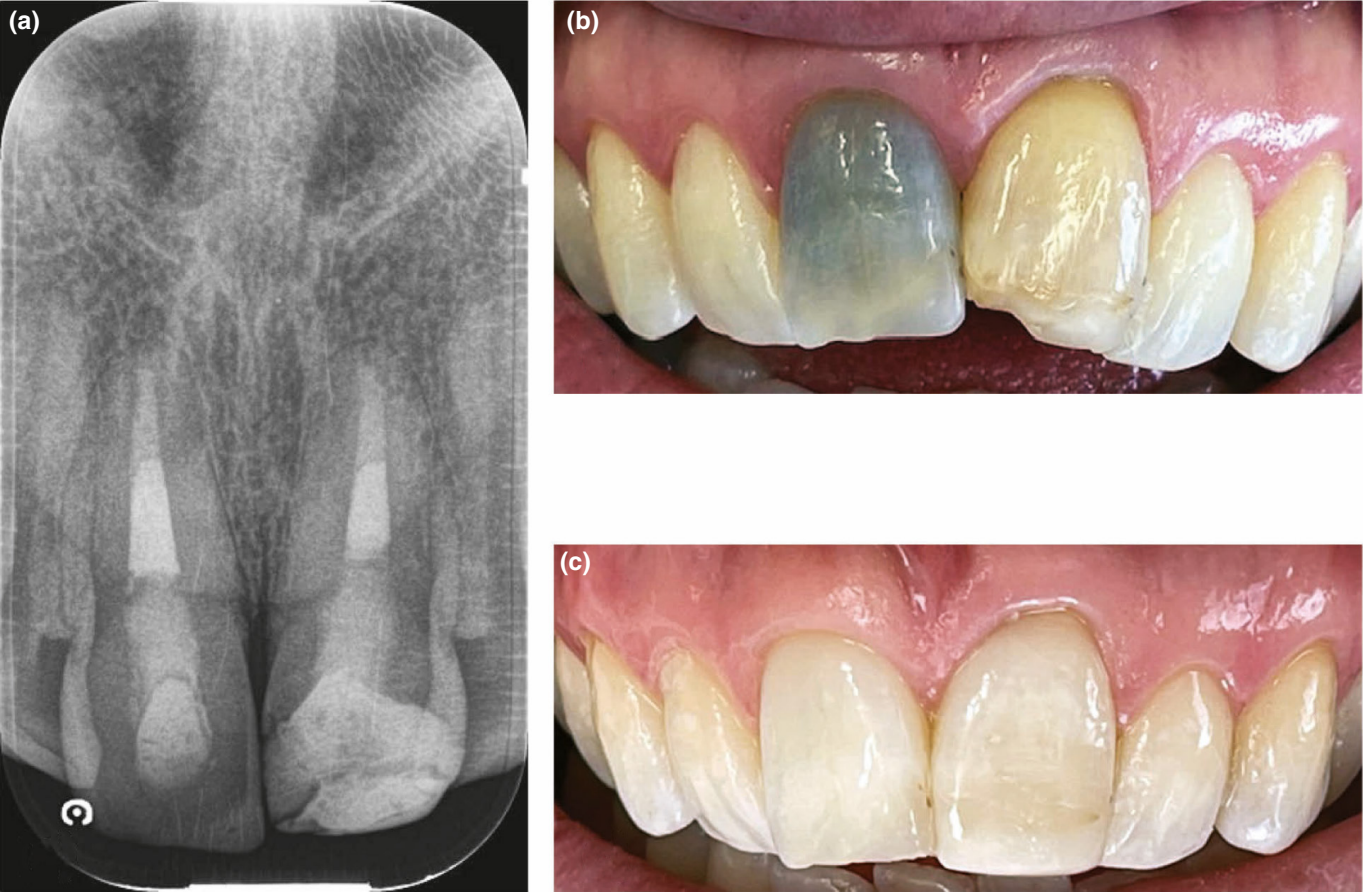
(a) A preoperative radiograph of the maxillary central incisors that are root filled with no evidence of pathosis. (b) A photograph of the right maxillary central incisor with a blackish discolouration. The left maxillary central incisor has a brownish discolouration. Bleaching was done with the walking bleach technique using 35% hydrogen peroxide (Opalescence, Ultradent Products. Inc.). (c) A post‐treatment photograph. The resin composite restoration in the left central maxillary incisor has been replaced. The right central maxillary central incisor was bleached in one appointment with the left central incisor requiring three appointments. There is a slight disparity between the colour of the two bleached teeth

**FIGURE 6:**
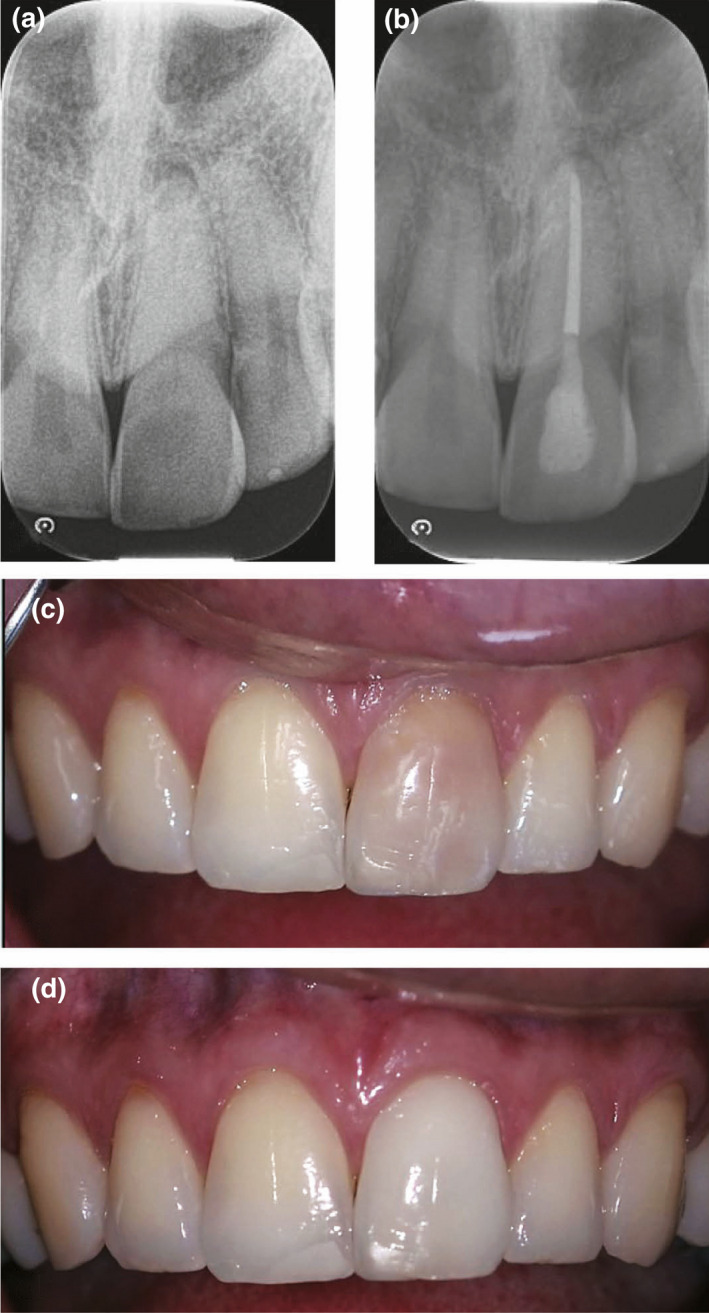
(a) A preoperative radiograph showing a left maxillary central incisor calcified canal with an obliterated canal and a periapical radiolucency. (b) A periapical radiograph of the tooth root filled. (c) A clinical radiograph revealing a brownish discolouration. (d) A clinical photograph of the tooth treated in three appointments using the bleaching protocol as described in Figure 5

Sodium perborate mixed with water is the most used technique for internal bleaching of root filled teeth. Alternatively, enhancement with 30%–35% hydrogen peroxide has been described (Abbott & Heah, 2009; Boksman et al., 1983; Nutting & Poe 1963,) but is less commonly used due to concerns with invasive cervical resorption. The protocol for the walking bleach technique is outlined in Table 4 and elaborated below. Cases using different bleaching agents are depicted in Figures 4, 5, 6.

**TABLE 4:** Walking bleach protocol

Step	Process	Considerations
1	Patient discussion	Informed consent. Discussion of options and risks, e.g. potential for discolouration, mix‐match in colour with adjacent teeth, invasive cervical resorption.
2	Clinical and radiographic assessment	Examination of defective restorations and/or caries. The presence of an adequate root filling is mandatory with no evidence of pathosis or recently root filled. Photographs prior to bleaching.
3	Access	Under rubber dam isolation, the access cavity needs to remove all restorative materials and root filling material, so a clean tooth substance is visualized including the pulp horns. Do not remove discoloured dentine.
4	Coronal seal of the root filling	The root filling should be removed below the level of the gingiva. If the root also requires bleaching an additional 2–3 mm of gutta‐percha should be removed. The root filling should be covered with a barrier of glass–ionomer cement (e.g. Vitrebond), or 2 mm of Cavit or IRM.
5	Refinement and finishing of the access cavity	The smear layer can be removed with a 10‐s rinse of phosphoric acid etch is controversial the smear layer may not be required to be removed. Desiccating the dentine with air and ethanol could be advantageous.
6	Bleaching material	The pulp space is filled with a thick mix of sodium perborate and water which can be condensed with an amalgam carrier or a Buchannan plugger. Excess water can be removed with cotton pellets but leaving the mix wet.
7	Provisional restoration	A 2–3 mm layer of Cavit or IRM cement is placed and pressed into undercuts and towards a clean cavo‐surface margin.
8	Bleaching time	The patient should return in 3–7 days to have more bleaching material placed. A total of one to four procedures may be required until the tooth is slightly lighter than the adjacent teeth.
9	Final restoration	The access cavity is restored with an acid‐etch composite restoration placed 1–3 weeks after the last bleaching appointment. A post‐bleaching photograph and periapical radiograph is recommended.

Note

Adapted from Dahl et al., 2019.

#### Steps 1–2: Patient discussion, clinical and radiographic assessment

A diagnosis of the aetiology of the discolouration is essential as well as a discussion of the patient's aesthetic expectations. A periapical radiograph indicating the likely absence of endodontic pathosis and an adequate root filling is mandatory prior to discussing bleaching. The patient should be informed that bleaching outcomes are not predictable and that complete restoration of colour is not always possible (Barateri et al., 1995; Rotstein, 2019). In fact, due to rebound in colour, the aim is for the bleached tooth to be lighter than the adjacent teeth (Attin et al., 2003; Plotino et al., 2008). Defective restorations need to be replaced prior to bleaching and there is the risk of post‐bleach mismatch in colour as restorations will not lighten with the bleaching procedure (Greenwall‐Cohen & Greenwall, 2019). The amount of remaining tooth structure could dictate alternative treatment options.

#### Steps 3: Access

Under rubber dam, the pulp space is endodontically accessed to identify the mesial and distal pulpal horns to remove potential residual necrotic tissue that may be a cause of the discolouration (Plotino et al., 2008). Visualization should be enhanced by magnification and illumination with either loupes or use of an operating microscope.

#### Step 4: Coronal seal of the root filling

The sealing barrier should be placed 1 mm coronally to the CEJ (Rotstein et al., 1992) and removal of the existing root filling may be required. The level of the barrier can be determined by internal measurements to validate the placement of the intra‐canal seal to match periodontal probing of the epithelial attachment at the mesial, distal, labial and palatal aspects of the tooth (Plotino et al., 2008). Therefore, teeth that have a gingival restoration may allow bleaching agents to be placed deeper than teeth with normal gingival architecture where the sealing barrier placed at the CEJ has the added advantage of minimizing the risk of invasive cervical resorption and damage to the periodontium (Steiner & West, 1994). A barrier of at least 2 mm is recommended to be placed on the root filling to prevent penetration of the bleaching materials into the root filling (Costas & Wong, 1991; Hansen‐Bayless & Davis, 1992; Smith et al., 1992). Barrier materials have included GIC‐based liners (e.g. Vitrebond, Espe 3 M), Cavit and IRM with Cavit having a superior seal to IRM which releases eugenol and can exacerbate the discolouration (Hansen‐Bayless & Davis, 1992). Cavit is easily removed and GIC‐based liners can be left *in situ* at the time of the final restoration (Setzer, 2020).

#### Step 5: Refinement and finishing of the access cavity

A final rinse with 1%–3% sodium hypochlorite for cleanliness (Attin & Kielbassa, 1995; Attin et al., 2003) is recommended or alternatively alcohol to reduce surface tension as dehydrated dentine may allow greater penetration of the bleach into the dentine. The use of 37% phosphoric acid to allow greater penetration of bleach (Beer, 1995; Hulsmann, 1993) is controversial as studies have shown no improvement in bleaching outcomes when the smear layer was not removed (Casey et al., 1989; Horn et al., 1998). The use of acidic agents as pre‐treatment of dentine may have adverse effects on the periodontium (Fuss et al., 1998). Therefore, removing the smear layer could have a deleterious effect (Attin et al., 2003).

#### Step 6: Bleaching materials

Sodium perborate (tetrahydrate) mixed with distilled water is commonly used and recommended in recent textbooks (Dahl et al., 2019; Setzer, 2020). In jurisdictions where sodium perborate is prohibited 18% carbamide peroxide can be used instead of sodium perborate (Kelleher, 2014). If discolouration is severe, water can be replaced with 3% hydrogen peroxide (Nutting and Poe, 1963). Some authors have advocated against the use of 30%–35% hydrogen peroxide due to possible risks of cervical resorption and damage to the periodontium (Friedmann et al., 1988; Kinomoto et al., 2001). However, others have used 35% hydrogen peroxide without development of cervical resorptions over 5‐year follow‐up reviews (Abbott & Heah, 2009). More rapid bleaching outcomes can be achieved with hydrogen peroxide, but similar results are achieved if sodium hydroxide is not used within short timeframes of 2 weeks with sodium and carbamide peroxide (Ari & Üngör, 2002; Holmstrup et al., 1988; Rotstein et al., 1991a, 1993; Vachon et al., 1998).

#### Step 7–8: Provisional restorations and bleaching time

Provisional seal/restoration is best achieved with more durable materials such as resin composite. Temporary materials have been shown to be inadequate (Waite et al., 1998) and loss of the provisional restoration would allow recontamination of the pulp space. Patients should be advised to monitor the colour change to minimize the risk of ‘over‐bleaching’ (Attin et al., 2003). There is the risk that a tooth can substantially lighten compared to adjacent teeth. The goal is to achieve a slightly lighter tooth than the adjacent teeth as there is likely to be some reversion of colour over time.

#### Step 9: Final restoration

A final restoration with an acid‐etch resin composite following bleaching should be deferred for 1–3 weeks (Cavalli et al., 2001; Shinohara et al., 2001, 2004; Unlo et al., 2008) as adhesion of composite and glass–ionomer cement to enamel and dentine is temporarily reduced following bleaching (Dishman et al., 1994; Garcia‐Godoy et al., 1993; Josey et al., 1996; Murchison et al., 1992; Swift & Perdigão, 1998; Titley et al., 1988, 1989, 1992; Toko & Hisamitsu, 1993). This is because remnants of peroxide and free oxygen have been shown to inhibit polymerization (Dishman et al., 1994; Torneck et al., 1990). It is not likely that change in the enamel structure interferes with composite adhesion (Ruse et al., 1990; Torneck et al., 1990). However, the appearance of composite tags in the hybrid layer in bleached enamel is less regular and distinct than in unbleached enamel (Titley et al., 1991). This may be why access cavities restored with composite can be associated with marginal leakage (Barkhordar et al., 1997). Several suggestions to negate the influence of hydrogen peroxide effects on bleached tooth structure are using dehydrating agents such as 80% alcohol or acetone‐containing adhesives (Barghi & Godwin, 1994; Kalili et al., 1993), the application of sodium hypochlorite to dissolve the remnants peroxide (Attin & Kielbassa, 1995; Rotstein et al., 1993), catalases (Rotstein, 1993), the antioxidant sodium ascorbate (Kaya et al., 2008; Lai et al., 2002) and alpha‐tocopherol (Whang & Shin, 2015). Optimal bonding to bleached enamel and dentine is restored after 3 weeks (Cavalli et al., 2001; Shinohara et al., 2001). In the interim period, calcium hydroxide placed in the access cavity may buffer the acidic pH that could be present after bleaching (Baratieri et al., 1995; Kehoe, 1987). Calcium hydroxide following bleaching does not interfere with the adhesion of the composite restoration (Demarco et al., 2001).

A post‐operative radiograph after bleaching and regular follow‐up radiographs is advised by the European Society of Endodontology (2006).

### Inside–outside closed bleaching

This approach combines the walking bleach technique with a single‐tooth external tray bleach to speed up the bleaching process and potentially reduce the number of appointments required to lighten the colour of the tooth (Haywood & DiAngelis, 2010). Considering most bleaching protocols are completed in one to three visits this additional step involving external bleaching may not be necessary.

### Inside–outside open bleaching

This approach allows the bleaching agent to be externally as well as inside the pulp chamber simultaneously (Liebenberg, 1997; Poyser et al., 2004; Settembrini et al., 1997) and involves leaving the access cavity open and protecting the root filling with a base. The patient regularly applies the bleaching agent (10% carbamide peroxide) with a syringe into the access cavity and externally into a tray every 4–6 h with the patient reviewed after 2–3 days to assess the change in discolouration (Greenwall‐Cohen & Greenwall, 2019). There is the potential for debris to accumulate into the access cavity. The advantages of both inside–outside approaches are not clear considering the well documented efficacy of the walking bleach technique. However, many authors advocate for this technique and state there is no evidence to associate the inside–outside bleaching protocol where low concentrations of hydrogen peroxide are used without heat with complications associated with the walking bleach technique when higher concentrations of hydrogen peroxide may be used (Leith et al., 2009).

### Novel techniques – Lasers

Studies have shown that the use of lasers enhances the bleaching process in the walking bleach technique when sodium perborate and a Nd:YAG laser irradiation is used (Sağlam et al., 2015). However, a recent study has shown that while laser irradiation can accelerate the bleaching process, the final results are not different to control groups without laser irradiation irrespective of the laser power settings (Papadopoulos et al., 2021). Refer to Table 3.

### Novel techniques – Cold atmospheric plasma

A recent case report has used cold atmospheric plasma applied into the access cavity of a root filled tooth without the use of conventional hydrogen peroxide bleaching agents (Pavelić et al., 2020). Cold atmospheric plasma is obtained from dielectric barrier discharge where the glass electrode functions as the primary electrode and the tooth as the secondary electrode. Therefore, more sophisticated equipment is required. The authors state that the differences between the standard walking bleach technique and the cold atmospheric plasma approach is that a root filling, protective cervical barrier and bleaching agent are necessary, and resorption could still be a possible risk. In the presented report the tooth was root filled.

## PROGNOSIS FOR BLEACHING EFFICACY

Table 2 sets out selected studies that have investigated bleaching efficacy. Many studies attest to the immediate efficacy of bleaching to improve the discolouration with lightening using a variety of different bleaching protocols (Abbott & Heah, 2009; Amato et al., 2018; Anitua et al., 1990; Brown, 1965; Holmstrup et al., 1988; Howell, 1981; Waterhouse & Nunn, 1996). Studies have also shown colour regression with time (Abbott & Heah, 2009; Brown, 1965; Feiglin, 1987; Friedman et al., 1988; Waterhouse & Nunn, 1996). Friedman et al. (1988) reported most failures in the aesthetic outcome occurred 2–8 years post‐bleaching. Colour failures were also associated with poor final restorations (Abbott & Heah, 2009; Chandra & Chawla, 1972). It was also shown that severely discoloured teeth are more difficult to bleach (Brown, 1965). It is reported that light yellow and grey discolourations are associated with better colour improvement than dark yellow and black teeth (Abbott & Heah, 2009). Studies have shown that there is high satisfaction after nonvital bleaching in anterior discoloured teeth (Gupta & Saxena, 2014). In that study, 87.8% of patients were highly satisfied with the bleaching outcome and only 4.9% of patients not satisfied. However, their concerns included the unpredictability of the final shade and potential for colour regression. The high level of satisfaction may also be due to the fact that these were intact teeth that had a traumatic injury. Similar satisfaction rates (87.1% good and 12.9% acceptable) were reported by Abbot and Heah (2009).

## CONTRAINDICATIONS TO BLEACHING OF ENDODONTICALLY TREATED TEETH

Teeth with extensive restorations may not respond as well to bleaching (Glockner et al., 1999; Howell, 1980). Intracoronal bleaching is not indicated unless pulp pathosis is evident (Rotstein, 2019).

## COMPLICATIONS AND RISKS

Tooth bleaching can have adverse risks on hard and soft dental tissues including external cervical resorption, adverse effects on adhesive bonding systems and dental material solubility (Anderson et al., 1999).

### External cervical root resorption

**FIGURE 7:**
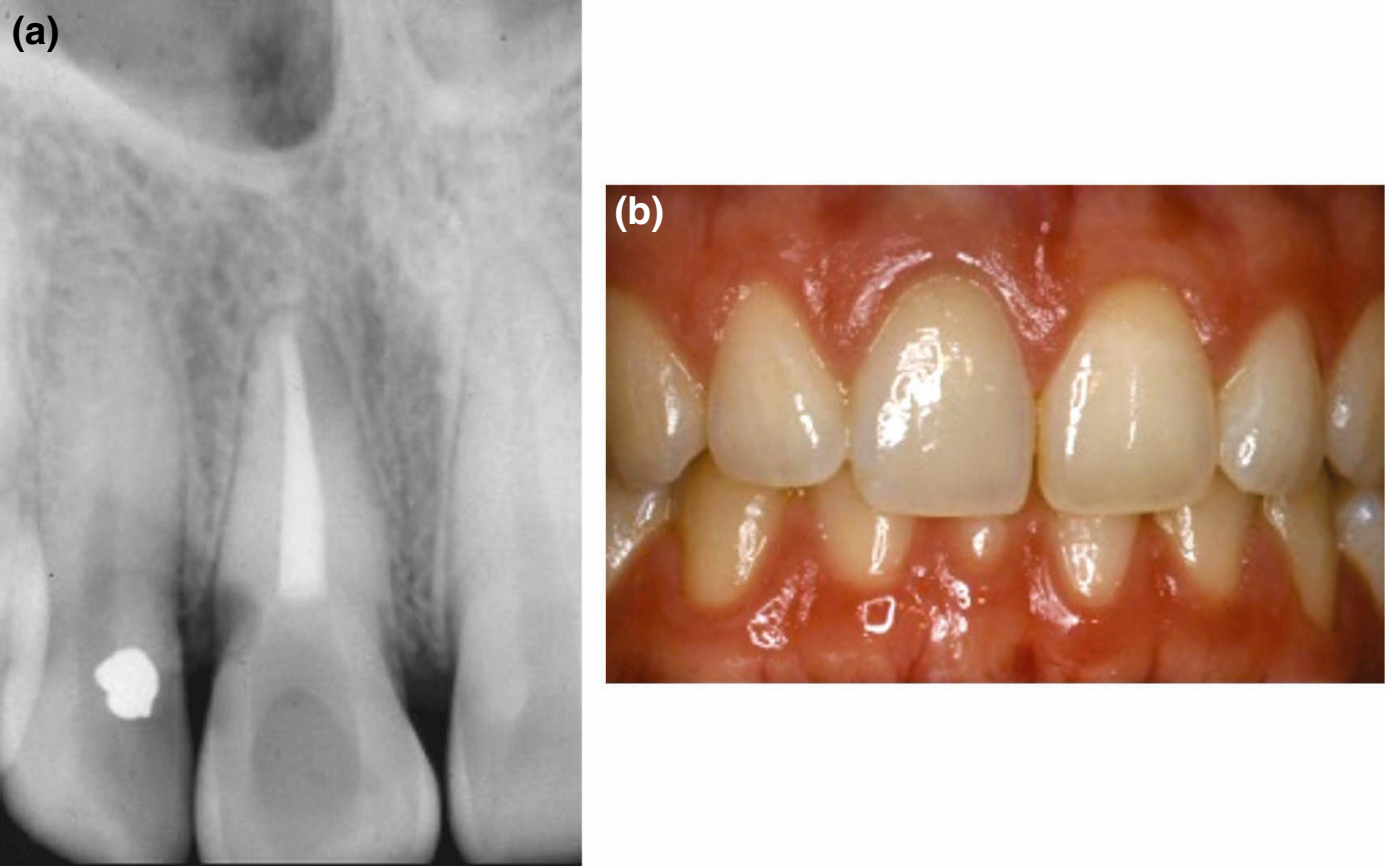
(a) A clinical photograph of the bleached left maxillary central incisor that had been bleached with the walking bleach technique with unknown medicaments. (b) A periapical radiograph revealing external cervical root resorption. Note the absence of any intracoronal barrier and large access cavity preparation. Courtesy of Professor Geoffrey Heithersay

External cervical root resorption is a serious complication of bleaching with peroxide compounds that can result in tooth loss. External cervical resorption has been reported in 6%–8% of cases which used 35% hydrogen peroxide and 18%–25% if the hydrogen peroxide was heat activated (Cvek & Lindvall, 1985; Friedman, 1997; Friedman et al., 1988; Harrington & Natkin, 1979; Heithersay et al., 1994; Lado et al., 1983; Rotstein et al., 1991b). Predisposing factors include cementum deficiency exposing dentine, a periodontal ligament injury and prior trauma (Baratieri et al., 1995; Esberard et al., 2007; Friedman et al., 1988; Harrington & Natkin, 1979; Koulaouzidou et al., 1996; Tredwin et al., 2006; Trope, 1997). Approximately 10% of teeth have an incomplete CEJ that exposes unprotected underlying dentine (Ten Cate, 1985). Hydrogen peroxide can penetrate to the cervical area of teeth and these areas of defects in CEJ morphology (Kopp, 1973; Neuvald & Consolaro, 2000; Rotstein et al., 1991b). Dental trauma can also damage the CEJ exposing underlying dentine (Madison & Walton, 1990; Montgomery, 1984). Free oxygen radicals that are the product of the bleaching process can breakdown collagen and hyaluronic acid which may be a pathological mechanism for resorption (Dahlstrom et al., 1997). These oxidizing agents can induce dentine protein denaturation (Lado et al., 1983) because of changes in pH (Demarco et al., 2001; Gimlin & Schindler, 1990; Montgomery, 1984) and heat (Freccia et al., 1982; Harrington & Natkin, 1979; Madison & Walton, 1990). Therefore, the application of heat and specifically the thermocatalytic technique in bleaching procedures is no longer advised (Setzer, 2020). A tooth that developed external invasive cervical resorption following trauma and bleaching is shown in Figure 7.

Inflammation may also be implicated in the aetiology of external cervical resorption (Trope, 1977). However, Heithersay (2004) argued that invasive cervical resorption was not activated by sulcular microorganisms and suggested the aetiology may involve a type of benign proliferative fibrovascular or fibro‐osseous disorder in which microorganisms have no pathogenic role but may become secondary invaders. It is thought that hydrogen peroxide compounds and free radicals must reach the periodontal tissues through the dentinal tubules and defects in cementum (Newton & Hayes, 2020; Wang & Hume, 1988). Significant increases in the levels of bone resorption markers RANK‐L and IL‐I ß were found in gingival crevicular fluid of teeth treated with walking bleach where the bleaching agent was 35% hydrogen peroxide or 37% carbamide peroxide (Bersezio et al., 2018b). Further study by the same group revealed that these inflammatory mediators persisted for 3 months post‐bleaching (Bersezio et al., 2020). This is a potential sign of harmful effects from bleaching procedures.

The use of sodium perborate instead of hydrogen peroxide may be a safer alternative (Madison & Walton, 1990). Sodium perborate mixed with water is less toxic to PDL cells than 30% hydrogen peroxide or sodium perborate mixed with 30% hydrogen peroxide (Kinomoto et al., 2001).

Others advocate the use of carbamide peroxide (Lim et al., 2004). The use of 35% carbamide peroxide has lower levels of extraradicular diffusion than comparable concentrations of hydrogen peroxide. Carbamide peroxide results in an alkaline pH in the tooth as it breaks down into ammonia which has a reduced etching effect. The dissociation of carbamide peroxide is equivalent to 12% hydrogen peroxide (Lee et al., 2004). However, carbamide peroxide is most often used for external bleaching.

Step 4 of the walking bleach technique advocates for a 2‐mm protective barrier over the gutta‐percha to the level of the CEJ to prevent penetration of hydrogen peroxide (Rotstein et al., 1992b). Heithersay et al. (1994) in a study where the walking bleach technique employed a thermocatalytic approach and 30% hydrogen peroxide with no coronal protective barrier over the gutta‐percha/AH26 root canal filling for 204 teeth reported an incidence of invasive cervical resorption of just 1.96%. In that study, for 84.37% of the teeth, the root filling was either at the level of the CEG or above. Only 18.63% of the teeth was the root filling below the CEJ. The follow‐up range was 1–19 years with a median of 4 years. The four afflicted teeth also had a history of a traumatic injury. Abbott &Yeah (2009) employed a walking bleach protocol with 35% hydrogen peroxide mixed with sodium perborate in a study of 255 teeth where a cervical seal of 2.5 mm of Cavit and reported no teeth developed external cervical resorption with a follow‐up of 6 months to 5 years. Current thinking suggests that the risk of external cervical resorption is a factor of the concentration of the hydrogen peroxide, whether a base/seal was placed to cover the root filling and if the action of hydrogen peroxide has been enhanced by heat. The studies by Heithersay et al. (1994) and Abbott and Heah (2009) where there was a low to nil risk of external cervical resorption respectively, suggest other factors are also involved. The prior association with trauma is identified (Heithersay et al., 1994). However, the mechanisms by which intracoronal bleaching is associated with external cervical resorptions are poorly understood (Attin et al., 2003; Patel et al., 2009). Hydrogen peroxide products placed into the access cavity diffuse through dentinal tubules and enamel to reach the external surfaces of the tooth and also the periodontal tissues (Palo & Bonetti‐Filho, 2012). Therefore, placement of a base/seal is advised (Oliviera et al., 2003). More controversially, is the recommendation to remove gutta‐percha to bleach the root in cases where root discolouration is visible through the gingival tissues. It is reasonable to expect a higher risk of invasive cervical resorption in these cases especially considering the known defects in the CEJ in some teeth heightening the risk association.

All the studies in Table 5 investigated the association of bleaching with invasive cervical resorption with the exception of Lise et al. (2018) that used 8%–10% carbamide peroxide, used concentrations of hydrogen peroxide that are no longer legal in the European Union (European Union produced a Cosmetic Directive [2011/84/EU]). Mavridou et al. (2017) in a study of 337 teeth with external cervical resorption reported an incidence 1% of cases where bleaching was identified as the sole predisposing factor. Unfortunately, this study did not identify the bleaching protocols used. However, as it was conducted in Europe in 2010–2015 it is likely to be in accordance with the European directive where the concentration of hydrogen peroxide products was legislated. Importantly, studies have shown that external resorption is associated with patients of a young age who have undergone bleaching (Abou‐Rass, 1998; Aldecoa & Mayordomo, 1992; Anitua et al., 1990; Glockner et al., 1999; Harrington & Natkin, 1979; Heithersay et al., 1994; Holmstrup et al., 1988; Lado et al., 1983). Also of significance, is that Amato et al. (2018) in a study of 40 teeth followed for 40 years using 10% carbamide peroxide with gutta‐percha covered with zinc oxide eugenol reported no incidence of external cervical resorption.

**TABLE 5:** Incidence of cervical resorption following internal bleaching in endodontically treated teeth in clinical studies and case series

Reference	Number of teeth	Bleaching agent	Cases of Cervical resorption	Age of patients	Cervical seal	Trauma	Heat	Review (years)
Clinical studies								
Friedman et al. (1988)	58	(a)TCC: 30% H_2_O_2_ (b)WBT: 30% H_2_O_2_ (c) TCC + WBT: 30% H_2_O_2_	1 1 2 (6.9% overall)	24 18 14	No No No	No No No	Yes No Yes	8
Holmstrup et al. (1988)	69	WBT: SP + water	None	Unknown	Yes	Yes	Yes	3
Anitua et al. (1990)	258	WBT: SP + 110 vol H_2_O_2_	None	—	—	—	—	4
Aldecoa and Mayordomo (1992)	258	WBT	None	—	—	—	—	6
Heithersay et al. (1994)	204	TCC: 30% H_2_O_2_ followed by WBT: 30% H_2_O_2_	4 (1.96%)	1:10–15 3: 16–20	No No	Yes Yes	Yes Yes	1–19 Median 4
Abou‐Rass (1998)	112	WBT SP + 30% H_2_O_2_	None	—	—	—	—	5–15
Glockner et al. (1999)	86	WBT: SP + 30% H_2_O_2_	None	Unknown	Yes			4–6
Amato et al. (2006)	35	Mixture of SP + 120 vol H_2_O_2_	None	7–30 Mean age 13.2	Yes	Majority 84%	Yes	16
Abbott and Heah (2009)	255	WBT 35% H_2_O_2_ + SP powder	None	<10–60 53.2% aged 11–20	Yes	Majority 58.8%	No	0.5–5
Amato et al. (2018)	60	EC + WBT 10% CP	None	18–35	Yes	35%	No	25
Lise et al. (2018)	17	9: SP + 20% H_2_O_2_ 8: I‐O 10% CP	None None	None None	Yes Yes	Unknown Unknown	No No	1 1
Case series								
Harrington and Natkin (1979)	7	TCC: 30% H_2_O_2_ followed by WBT: 30% H_2_O_2_	7 (100%)	15	No	Yes	No	2–7
Cvek and Lindvall (1985)	11	TCC: 30% H_2_O_2_ followed by WBT: 30% H_2_O_2_	11 (100%)	<21	No	Yes: 10 No: 1	Heat	1
Badole et al. (2013)	3	35% CP	0	19–35	Yes	Yes	No	0.4–1

Note

Adapted from Attin et al. (2003). However, case reports were not included due to potential of publication bias. Important to note bias in Harrington and Natkin (1979) and Cvek and Lindvall (1985) as specifically reporting cases of cervical resorption.

Abbreviations: CP, carbamide peroxide; EC, extra‐coronal; H_2_O_2_, hydrogen peroxide; I‐O, inside–outside technique; SP, sodium perborate; TTC, thermocatalytic; WBT, walking bleach technique.

The risk of external cervical resorption using modern bleaching procedures is likely to be lower than prior studies that used heat and higher concentrations of hydrogen peroxide (Newton & Hayes, 2020). Bleaching of root filled teeth with the walking bleach technique is considered a safe protocol with a low risk of invasive cervical resorption (Dahl et al. 2019; Newton & Hayes, 2020; Setzer, 2020).

Treatment of invasive cervical resorption is not specifically discussed in this review but affected teeth can be with the use of direct restorations (Friedmann, 1989; Heithersay, 1999, 2007; Meister et al., 1986). After debriding the resorptive defect it is recommended to treat the resorptive defect with 90% trichloroacetic acid to induce sterile necrosis of remaining resorptive tissue (Heithersay, 1999, 2007).

### Enamel and dentine damage

Many laboratory studies have reported changes in enamel microhardness and morphology (Bitter, 1998; Chng et al., 2005; Grazioli et al., 2018; Lopes et al., 2002; Murchison et al., 1992; Rotstein et al., 1992a, 1996; White et al., 2002; Zanolla et al., 2017) and in cementum (Tong et al., 1993; Zalkind et al., 1996). It has been proposed that peroxide components alter the ratio of organic to inorganic hard tissue compounds (Heling et al., 1995; Lewinstein et al., 1994; Powell & Bales, 1991; Tong et al., 1993). However, this has been found to be concentration‐dependent as 35% carbamide peroxide changed the inorganic composition of the enamel where lower concentrations of 10%–16% had no effect on enamel composition (Oltu & Gürgan, 2000). However, even high concentrations of 30% hydrogen peroxide and 30% hydrogen peroxide mixed with sodium perborate was associated with less morphological damage to the external surface of enamel than 37% phosphoric acid (Ernst et al., 1996). The clinical significance of this alteration in enamel composition and surface structure is not clear. This is more of a consideration for external bleaching and outside the scope of this review.

### Inhibition of adhesive bonding

As discussed in Step 9 final restoration with an acid etch adhesive restoration should be deferred for 1–3 weeks as polymerization and bonding strengths of composite resins to dentine and enamel are temporarily affected by the oxygen released during bleaching (Cavalli et al., 2001; Sinohara et al., 2001, 2004). This is important as failure of the final restoration can cause future discolouration.

### Gingival irritation

Gingival irritation is a greater concern for external bleaching. However, protection of the oral tissues with rubber dam is advised (Heithersay et al., 1994). The use of wedges and blockout materials such as Orabase or Opal Dam to properly seal the rubber dam to protect the gingival tissues is also advocated (Rotstein, 2019; Setzer, 2020).

### Regenerative endodontic and vital pulp therapy technique complications

**FIGURE 8:**
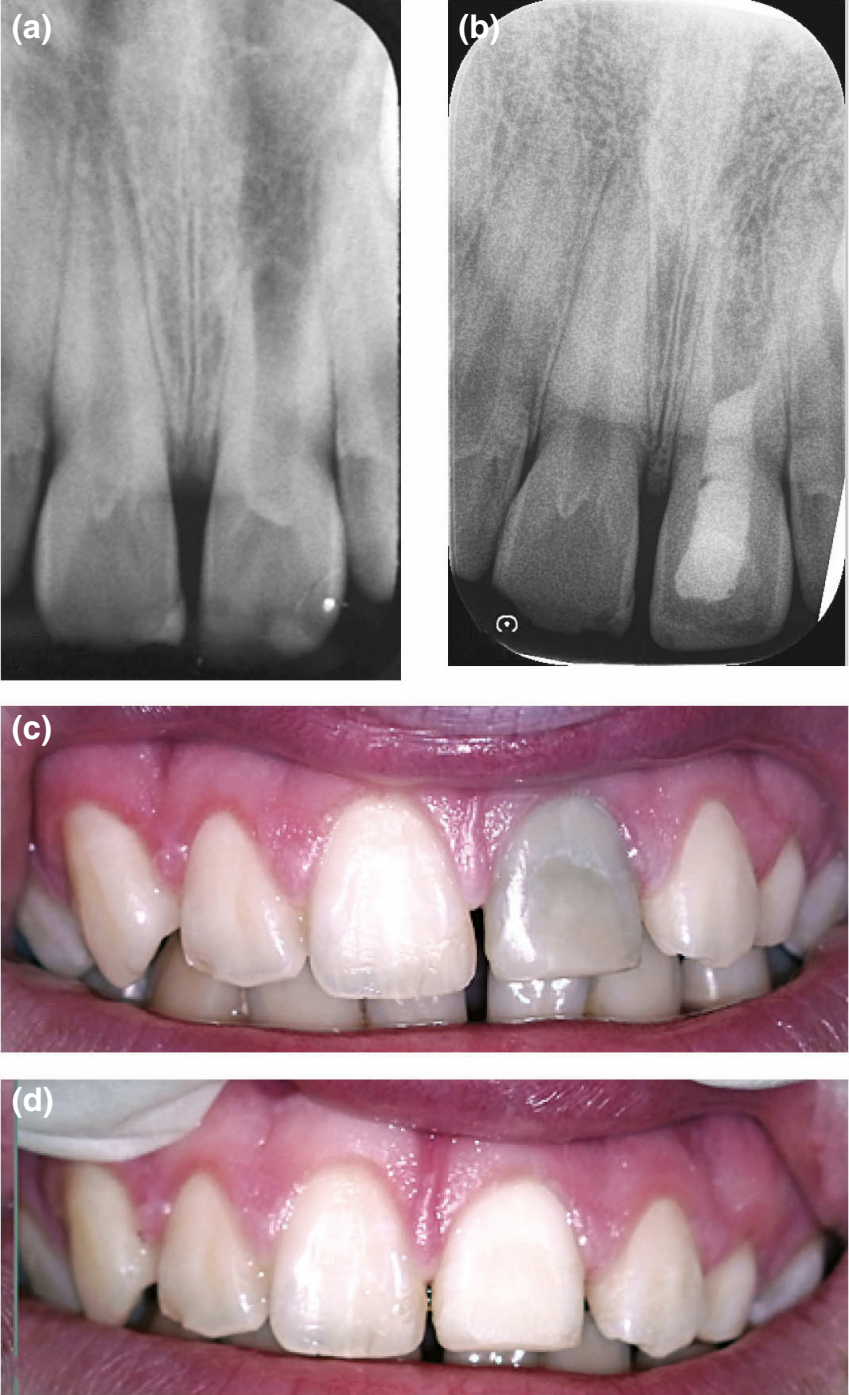
(a) A preoperative radiograph showing a left maxillary central incisor with interrupted root development indicating pulp necrosis. (b) The tooth as treated with regenerative endodontic protocols. (c) A photograph revealing blackish/greyish discolouration. (d) A photograph of the tooth after three bleaching appointments using the protocol described in Figure 5. Reprinted in part from (Fida et al., 2021) with permission

Tooth discolouration caused by TAPs can be reversed by the walking bleach technique with sodium perborate used as the bleaching agent (Kirchhoff et al., 2015). However, not all teeth treated with REPs that are bleached are successfully resolved with bleaching with sodium perborate mixed with 10% hydrogen peroxide (McTigue et al., 2013). Bleaching with 35% hydrogen peroxide of a discoloured tooth treated with REPs using TAPS and MTA is shown in Figure 8. Calcium hydroxide resulted in significantly less discolouration than TAP when used as an inter‐visit medicament (Nagata et al., 2014). Biodentine used as a coronal barrier may result in in less discolouration than MTA (Bakhtiar et al., 2017). A laboratory study also reported that Endocem resulted in less discolouration than MTA materials. Furthermore, removal of the MTA was more effective at improving the discolouration than internal bleaching with sodium perborate mixed with 3% hydrogen peroxide (Jang et al., 2013). Belobrov and Parashos (2011) also successfully bleached a vital discoloured traumatized incisor treated with a partial pulpotomy using white MTA. The tooth was bleached with sodium perborate mixed with saline.

## PROSTHODONTIC OPTIONS – RESIN COMPOSITE AND CERAMIC VENEERS, CROWNS

**FIGURE 9:**
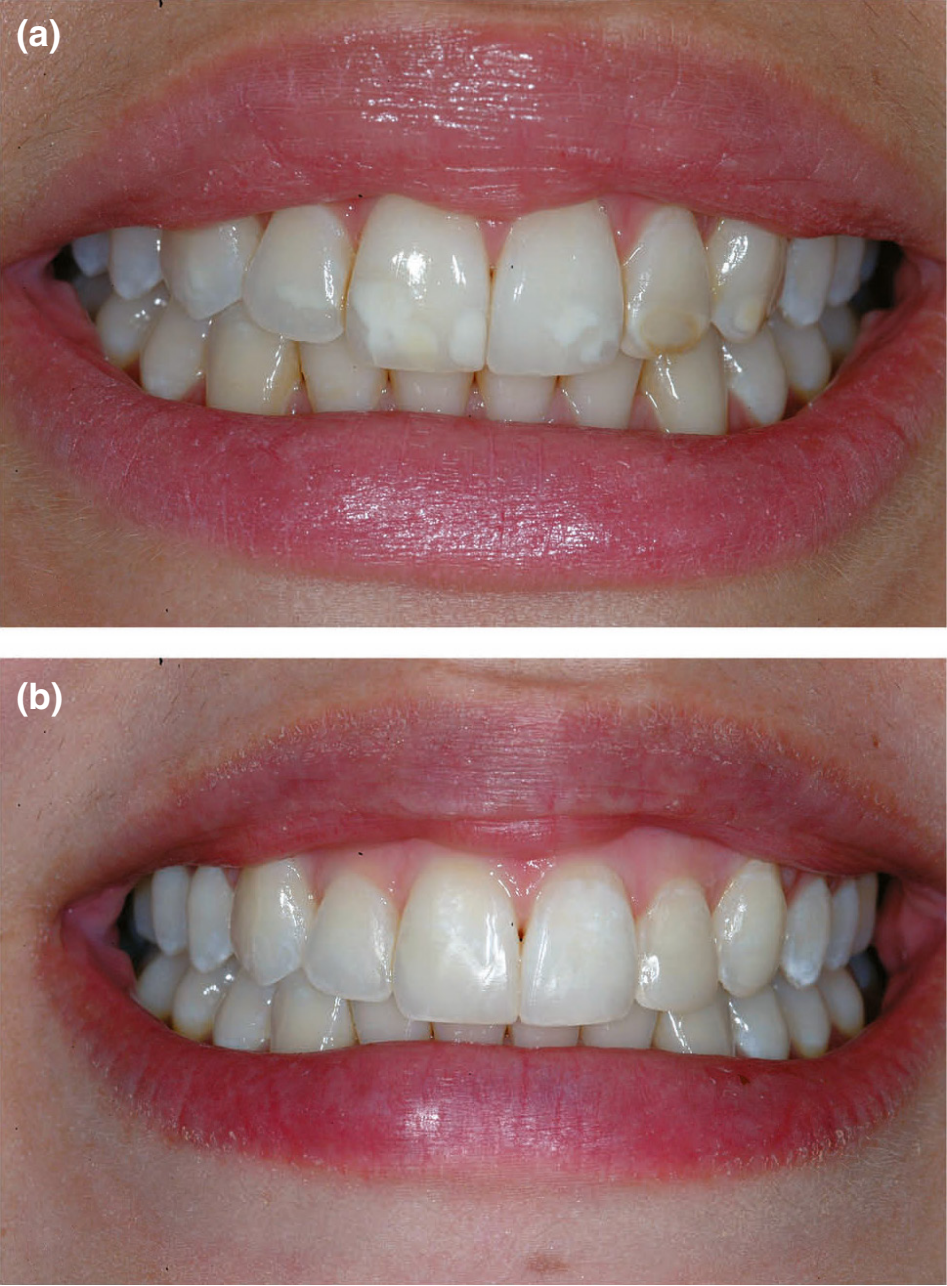
(a) A photograph of vital teeth with mild fluorosis (b). A post‐treatment photograph after management involved KTP laser activation and resin composites. Courtesy of Dr Mark Gervais

**FIGURE 10:**
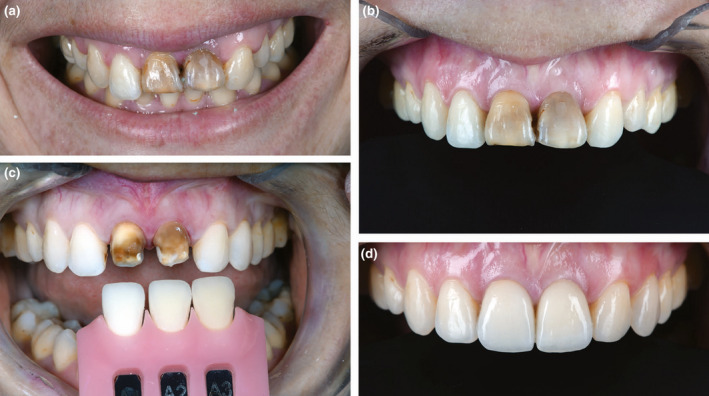
(a) A photograph of discoloured root filled maxillary central incisor which are inferior to the occlusal arch influencing the aesthetic appearance. (b) The teeth after improved oral hygiene. (c) Colour selection with the teeth prepared for porcelain veneers. (d) A photograph taken after cementation of the veneers. Courtesy of Dr Tony Rotondo

**FIGURE 11:**
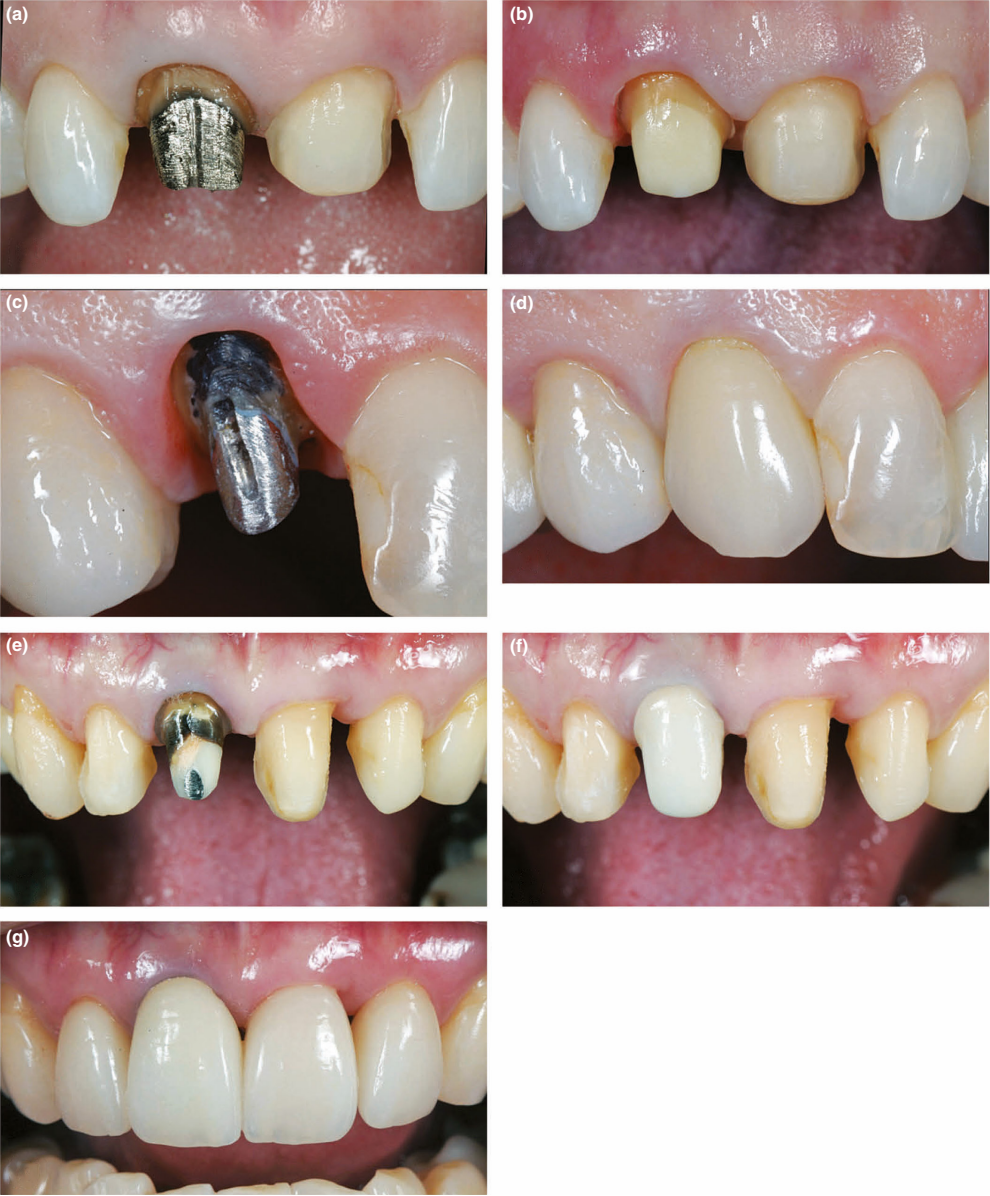
(a, b) Improving prosthodontic outcomes by removing metal posts. (c, d) Masking metal posts and discoloured teeth with a VMK crown. (e‐g) Masking a discoloured maxillary right central incisor with a dual crown approach using a YTP Zirconium crown which is then overlaid with a more translucent eMax crown. Courtesy of Dr Michael Mandikos

**FIGURE 12:**
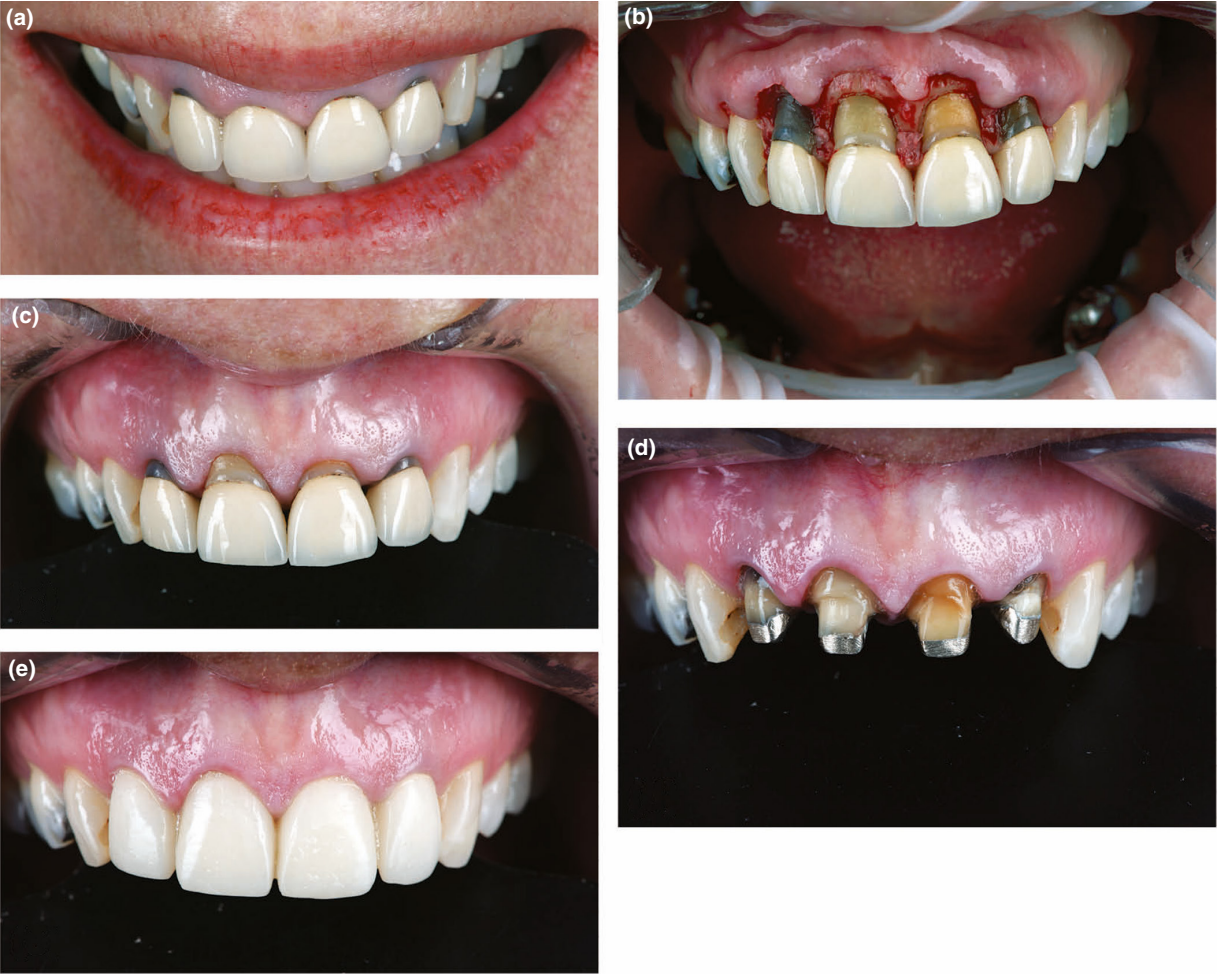
(a) A photograph of maxillary incisors revealing prior crown rehabilitation with short clinical crowns and gingival recession showing discoloured roots in the root filled teeth. (b) A photograph revealing crown lengthening so the appearance of the teeth can be improved. (c) A photograph of the new gingival position to allow teeth to have a more leasing height to width ratio. (d) A clinical photograph showing the teeth prepared to the new gingival position. (e) A post‐treatment photograph with porcelain crowns. Courtesy of Dr Tony Rotondo

Prior reviews have indicated that direct composite veneers, porcelain veneers and crowns are alternatives to bleaching (Greenwall‐Cohen & Greenwall, 2019) (Figures 9, 10, 11, 12). It is important that patients are made aware of the more extensive loss of tooth structure, expense, risks and complications including repair and replacement with time (Alani et al., 2015). Ceramic veneers had better outcomes than indirect composite veneers in terms of survival rate and the quality of the restorations over a 10‐year follow‐up (Gresnigt et al., 2019). A recent study reported leucite‐reinforced glass–ceramic crowns had a survival rate of 79.6% after 11–13 years with ceramic fractures responsible for most clinical failures (Zürcher et al., 2021). A systematic review and meta‐analysis of feldspathic porcelain and glass–ceramic laminate veneers reported failures associated with debonding, fracture/chipping, secondary caries and severe marginal discolouration (Morimoto et al., 2016). Direct composite resin veneers are more affordable, less invasive and easier to repair than indirect porcelain veneers but are more likely to sustain colour loss and over time. Furthermore, the aesthetic outcome of indirect porcelain veneers depends on the dentine adhesive and resin luting cements (Araujo & Perdigão, 2021). Consistent perfect results are difficult to achieve especially in the discoloured tooth because of the dark substrates of the crown and the root (Mandikos, 2021). Masking the dark substrate of the crown/and or post can be improved by removing metal posts or masking posts with composite opacifiers (Figure 11). Masking the discoloured tooth with zirconium copings can present technical issues and a higher incidence of chipping of feldspathic porcelain (Mandikos, 2021).

## PERIODONTAL CONSIDERATIONS – ROOT COLOUR, TOOTH SHAPE AND FORM

Patients perception of root colour, tooth shape and form can involve changing the aesthetic appearance of the tooth with periodontal surgery (Figure 12). This is a common procedure in the aesthetic zone (Marzadori et al., 2018). However, a recent systematic review reports complications of gingival rebound and whether biological width is re‐established (Al‐Sowygh, 2019).

## PERFECTIONISM AND QUALITY OF LIFE

Patients and dentist's expectations for aesthetic outcomes can be high. There is an increasing public awareness for lighter teeth and the word ‘Hollywood smile’ has entered our lexicon. This may be a consideration for the walking bleach technique where a lighter colour than the adjacent teeth is the desirable outcome due to potential rebound in colour. Pavicic et al. (2018) report that quality of life can be improved with bleaching. However, patient's personality traits and expectations should be considered in attaining treatment outcomes. While the treatment of discoloured teeth may involve an inter‐disciplinary approach, the perfect result may be unattainable, particularly for some patients with heightened expectations. The pursuit of perfection with porcelain may also not be predictable and maybe very dependent on the clinicians' technical capabilities which will be variable between different operators.

## THE EUROPEAN UNION AND THE LAW

Due to the European Union directive (2012) it is either not possible or there is reluctance by practitioners to bleach discoloured teeth in patients under the age of 18 (Trainor & Good, 2016; Walshaw et al., 2019). Despite many recent textbooks advocating the use of sodium perborate, in Europe 10% carbamide peroxide should be used to comply with the European Union cosmetic directive. This is because the Cosmetic directive allows for a maximum concentration of hydrogen peroxide of 6% where sodium perborate mixed with water releases 7% hydrogen peroxide as opposed to 3.5% release when carbamide peroxide is used as the bleaching agent. Furthermore, bleaching is prohibited for people under the age of 18 years. Unfortunately, as many teeth darken from dental trauma and children are generally the more at‐risk group to traumatic events. There is a concern that the directive may place dentists and patients at risk of choosing more invasive and expensive procedures. Dental bleaching has been shown to be a safe and effective means of lighten the colour of teeth and the risks of external cervical resorption with modern internal bleaching protocols involving lower concentrations of hydrogen peroxide agents may be lower than earlier studies (Newton & Hayes, 2020). Nonvital bleaching provides predictable and conservative lightening of teeth when compared to prosthodontic alternative treatment options. Further research is required.

## FUTURE DIRECTIONS

The increasing popularity of tooth whitening of vital teeth may see the use of bleaching agents that do not comply with legislation. Recently, in Europe, many tooth whitening products did not comply with a European Union regulation. The European Network of Official Cosmetic Control Laboratories (OCCL) reported that only 71% of tested materials complied. The European Directorate for the Quality of Medicines (EDQM) reported that most noncompliance issues related to products exceeding the regulated concentration of hydrogen peroxide, the presence of carcinogenic, mutagenic and toxic substances such as sodium perborate as well as labelling issues (Dentistry Today, 2019). In Australia, 50% of consumers purchase do‐it‐yourself whitening products at pharmacies and on‐line outlets. These products may not fully disclose product information and/or mislead consumers (Australian Dental Association, 2019). The use of nonvital bleaching is increasingly regulated in many international jurisdictions (Grum, 2020). However, in the USA, most hydrogen peroxide bleaching agents are considered cosmetics rather than drugs and hence do not require Federal Drug Administration (FDA) approval. The safety and regulatory issues are the responsibility of the manufacturers (Grum, 2020). Unfortunately, the European Directives primarily concerned with materials considered cosmetics also impacts on endodontically treated teeth and the concentration and type of materials used in walking bleach protocols. In particular, this presents legal and ethical concerns when bleaching discoloured teeth in younger patients (Kelleher, 2014). Walshaw et al. (2019) share these concerns for the paediatric setting where alternative therapies that involve tooth loss are considered even at an early age. It is likely with many practices emphasizing cosmetic dentistry, that adult patients will enhance their smile with prosthodontic and periodontal treatment when cosmetic approaches using bleaching agents fail to achieve a pleasing aesthetic result. It is also likely when more tooth destructive treatments are chosen, issues other than tooth colour such as tooth wear, prior restorations and gingival contour will influence treatment selection decision‐ making processes as bleaching approaches are generally safe and effective. The importance of informed consent and the risks and benefits of the alternative treatment protocols will remain paramount for patient's and dentists.

## CONCLUSION

Managing discoloured teeth involves a clear understanding and diagnosis of the aetiology of the discolouration and interdisciplinary management. There is commonly an association with trauma, developmental and lifestyle considerations.

Bleaching endodontically treated teeth can be considered a safe and effective protocol in the management of discoloured teeth. The risk of external cervical root resorption may be lower with the use of sodium perborate and a change from higher concentrations of hydrogen products. However, the association between bleaching and resorption remains unclear although there is likely to be a relation to prior trauma. It is prudent to avoid thermocatalytic approaches and to use a base/sealer as a protective barrier to cover the gutta‐percha.

It is likely, that there will be continued public interest in lightening discoloured teeth. It is important that the patient can make an informed choice.

## CONFLICTS OF INTEREST

The author has no conflicts of interest to declare.

## AUTHOR CONTRIBUTIONS

Bill Kahler was the sole author and responsible for the design and content of the manuscript.

## ETHICAL APPROVAL

The article is a review and did not involve human participants. It therefore complies with the 1964 Helsinki Declaration and its later amendments or comparable ethical standards.
